# Structural and Functional Characteristics of Potent Dioxygenase from *Moesziomyces aphidis*

**DOI:** 10.1021/jacsau.5c00456

**Published:** 2025-06-12

**Authors:** Lukas Schober, Jacek Plewka, Kanokkan Sriwaiyaphram, Björn Bielec, Astrid Schiefer, Thanyaporn Wongnate, Katarzyna Magiera-Mularz, Florian Rudroff, Margit Winkler

**Affiliations:** † Institute of Molecular Biotechnology, 27253 Graz University of Technology, NAWI Graz, Petersgasse 14, 8010 Graz, Austria; ‡ Department of Organic Chemistry, Faculty of Chemistry, 37799 Jagiellonian University, Gronostajowa 2, 30-387 Krakow, Poland; ◧ School of Biomolecular Science and Engineering, 423058 Vidyasirimedhi Institute of Science and Technology (VISTEC), Rayong 21210, Thailand; # Institute of Applied Synthetic Chemistry, TU Wien, Getreidemarkt 9, 1060 Wien, Austria; ⊥ 298883 Austrian Center of Industrial Biotechnology, Krenngasse 37, 8010 Graz, Austria

**Keywords:** protein structure, dioxygenase, alkene cleavage, vanillin, aldehyde

## Abstract

Enzymatic C=C double bond cleavage to give carbonyl-species is an emerging alternative to ozonolysis, or stoichiometric use of metal-oxidants. The substrate scope of 4-His Fe dioxygenases, however, appears to be restricted to aromatic compounds with a hydroxy group at the 4-position of the aromatic ring. In-depth structural and functional characterization is a prerequisite to understand and ultimately to extend the substrate scope of this family of enzymes. Herein, *Moesziomyces aphidis* DSM 70725 aromatic dioxygenase (*Map*ADO) is characterized through X-ray crystallography, biophysical as well as biochemical assays, substrate docking and mutagenesis. *Map*ADO features a seven-bladed β-propeller fold and a Fe^2+^ center coordinated by four histidine residues and shares a conserved structural motif with homologous enzymes despite low sequence identity (<38%). Fe^2+^ is tightly bound and present in the catalytically active oxidation state at ambient conditions. *Map*ADO is robust and retains activity for several freeze/thaw cycles. *Map*ADO’s interaction with ligands 4-hydroxybenzaldehyde, *ortho*-vanillin and vanillin indicate that hydrogen-bonding of the phenolic OH group is key to activity. Structural analysis and site-directed mutagenesis indicate that two key residues (Y136 and K169), and the substrate’s hydroxy group, are essential for accurately positioning the double bond toward the activated oxygen at the Fe center. *Map*ADO wild-type exhibits the highest reported activity for converting isoeugenol to vanillin (231 μmol min^–1^ mg^–1^).

## Graphical abstract

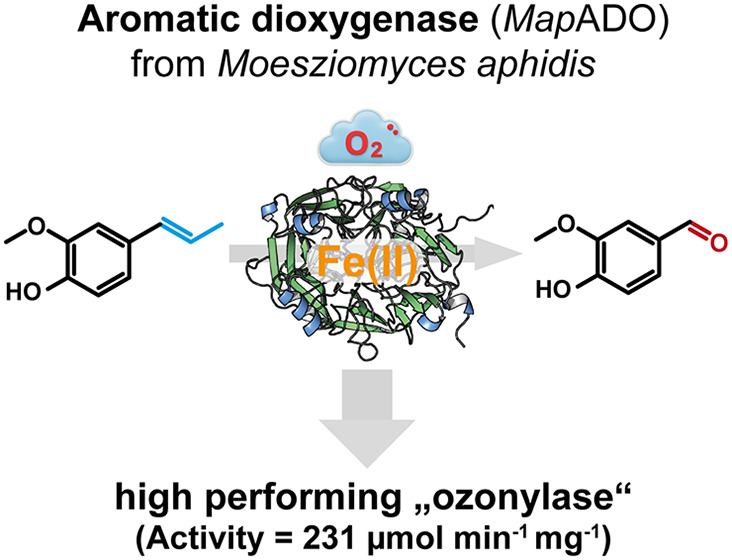

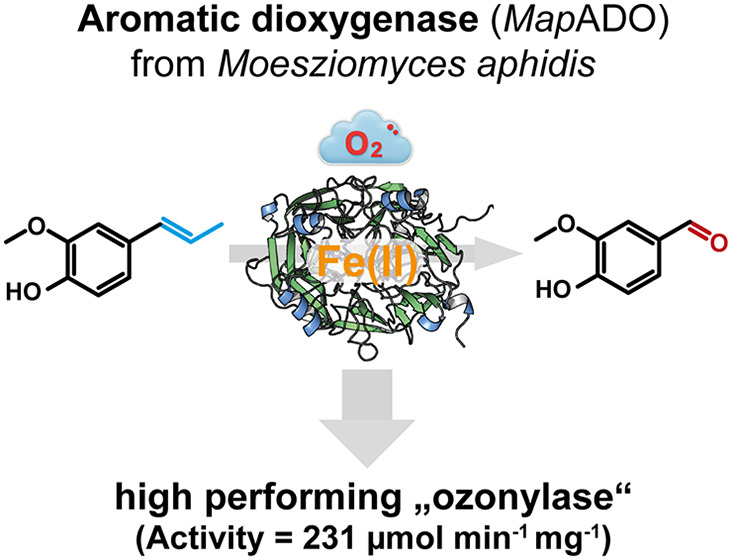

Carotenoid cleaving oxygenases (CCOs) are β-propeller fold enzymes. This versatile fold is found in a wide range of proteins and is associated with diverse functions, including catalysis, signal transduction, and molecular recognition.^1,2^

CCOs have attracted attention, especially for vanillin **1** synthesis from renewable resources, such as isoeugenol **1a** (Scheme 1), which can be derived from eugenol or lignin streams. CCOs catalyze the cleavage of C=C bonds also in other compounds, including carotenoids, stilbenes (e.g., resveratrol **3a**), and 4-vinylguaiacol.^3,4^ While ozonolysis is a common chemical method, it involves handling reactive and hazardous ozone gas.^5^ Alternatives, such as oxidation with hydrogen peroxide or molecular oxygen, require catalysts like vanadium but face limitations due to inefficiency, handling difficulties, and the generation of heavy metal pollutants and solvent waste.^6^

**Scheme 1:**
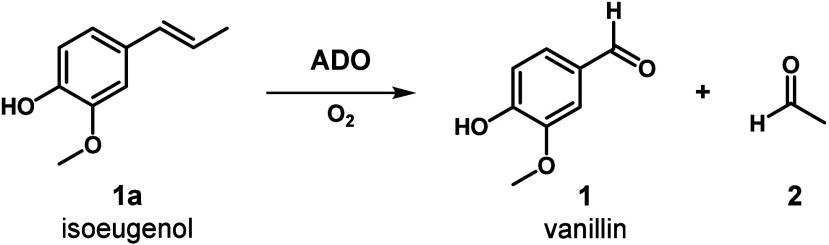
Alkene Cleaving Activity of Aromatic Dioxygenase (ADO)

Among the best-studied and most promising enzymes in this family are isoeugenol monooxygenase (IEM) from *Pseudomonas putida* IE27,^7^ and *Pseudomonas nitroreducens* Jin1,^8,9^ as well as Cso2 from *Caulobacter segnis* ATCC 21756.^10^ Similar proteins were termed aromatic dioxygenase (ADO),^11,12^ apocarotenoid^13^ or alkene cleaving oxygenase^14^ (ACO). These enzymes primarily act on aromatic compounds with a hydroxy group in 4-position and use a mononuclear Fe^2+^ iron that is embraced by four histidines. O_2_ is activated by this nonheme iron and reacts with the C–C double bond of a substrate, eventually leading to C–C bond scission and two carbonyl species as the reaction products (Scheme 1).

This study focuses on structural and functional characteristics of a yet uncharacterized protein from the yeast *Moesziomyces aphidis* (*Map*ADO). Phylogenetic sequence analysis reveals that *Map*ADO is most closely related to *Bf*RCO1 from *Botryotinia fuckeliana* and *Um*RCO1 from *Ustilago maydis* ^15^ (SI Figure S1). Here, we unveil *Map*ADO’s interactions with cocrystallized ligands. By analyzing the protein structure both in its native form and in complexes with vanillin **1**, 4-hydroxybenzaldehyde (HBA, **3**), and *ortho*-vanillin **5**, we uncover the nuances of ligand binding and coordination within *Map*ADO’s active site. Site directed mutagenesis, combined with activity assays were employed to identify essential residues. The insights have implications beyond basic science, informing the design of improved enzymes for industrial applications and potentially guiding the development of inhibitors targeting similar metalloenzymes in pathogenic organisms.

*Map*ADO (hypothetical protein PaG_05861) was efficiently produced in *E. coli*. Notably, the gene was only expressed when coding sequences for affinity tags, like a 6xHIS or strep tag, were included. Without these tags, the protein was undetectable in both soluble and insoluble fractions (SI Figure S2).

We tested alkene-cleavage activity of *Map*ADO through incubation with **1a** – a classical substrate for oxygenases (Scheme 1).^8,16,17^ The C=C double bond in the side-chain of **1a** was readily cleaved to give **1**. In comparison to ADO,^11^ higher conversion was obtained with 10-fold less biocatalyst at 5-fold higher substrate concentration (Table 1). Kinetic parameters were determined by spectrophotometric monitoring of **1** formation with purified ADO and *Map*ADO, respectively (Table 2) and show more than 5-fold faster reaction of *Map*ADO and higher affinity for **1a**. To the best of our knowledge, *Map*ADO exhibits the highest activity among enzymes reported in the literature, even though literature values are frequently based on single end-point measurements (SI TableS1).

**Table 1:** Resting Cell Biotransformation of 1a to 1^a^

enzyme	OD_600_	**1a** [mM]	Time [h]	Conversion [%]
ADO^11,16^	60	10	1	37
*Map*ADO	60	10	1	97
*Map*ADO	10	10	1	96
*Map*ADO	6	50	1	49
ADO	60	10	16	78
*Map*ADO	6	50	16	98

a T: 40°C, 2 vol% EtOH.

**Table 2:** Kinetic Data of Selected ADOs with Isoeugenol

enzyme	K_M_ [mM]	*V*_max_ [μmol min^–1^ mg^–1^]	*k*_cat_ [s^–1^]
ADO (*Tt*CCO)	2.240 ± 0.264	43 ± 0	44
*Map*ADO	0.118 ± 0.013	231 ± 2	238

In addition to **1a**, **3a** was cleaved to **3** and 3,5-dihydroxybenzaldehyde **4**, respectively (Scheme 2A, data not shown). The cleavage of the isoeugenol isomer 2-methoxy-6-prop-1-enylphenol (*ortho*-isoeugenol **5a**) would produce **5** as the reaction product, however, no **5** was produced (Scheme 2B).

**Scheme 2:**
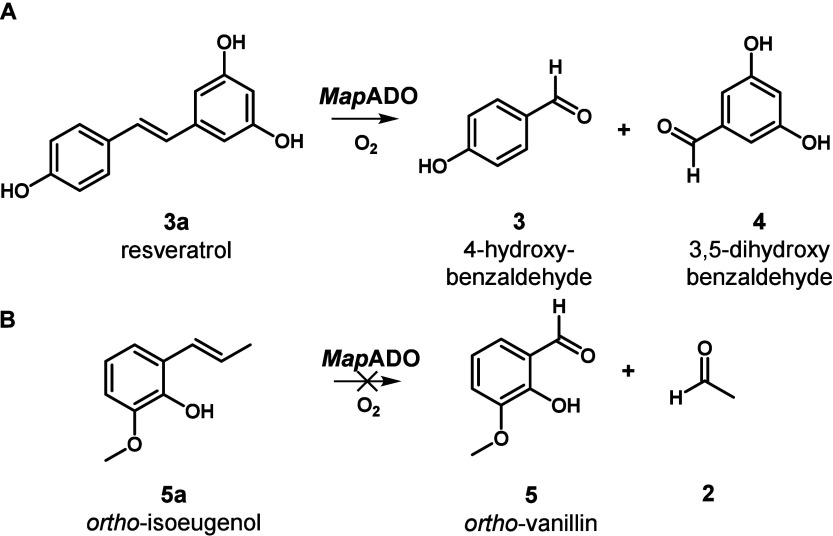
A: Oxidative Cleavage of Resveratrol Catalyzed by *Map*ADO, B: Oxidative Cleavage of *ortho*-Isoeugenol Catalyzed by *Map*ADO (not observed)

*Map*ADO’s thermal stability showed a 30% activity loss after 20 min at 40 °C and 90% at 45 °C, with no activity at higher temperatures (SI Figure S3). It retained activity at 30 °C across pH 7.0–9.0, but activity dropped sharply below pH 7.0, with only 40% activity at pH 6.0 and negligible activity at pH 5.5 or lower (SI Figure S4).

Tests with reducing agents (FeCl_2_, sodium dithionite, dithiothreitol, sodium ascorbate)^18^ revealed no improvement in activity, suggesting the presence of catalytically active Fe^2+^ under ambient conditions (SI Figure S5). Attempts to create apo-*Map*ADO using chelators (EDTA, EGTA) or Chelex resin did not significantly reduce activity, indicating strong iron binding (SI Figure S6).

Purified *Map*ADO, whole cells, and cell-free extracts (CFE) showed similar activity, with *Map*ADO retaining >90% activity in whole cells and CFE after five freeze–thaw cycles (SI Figure S7). To address substrate solubility, 2 vol % EtOH was selected as the standard cosolvent (SI Figure S8).

With *Map*ADO being a highly promising catalyst (Table 1), we proceeded to solve its structure. *Map*ADO features a seven-bladed β-propeller fold with an Fe^2+^ ion at the active site coordinated by H200, H251, H316, and H510 (Figure 1, SI Figure S9, TableS2). The electron density map confirmed iron coordination in an octahedral geometry, with acetate and water occupying the active site in the absence of reaction related ligands (PDB ID: 9G88). Compared to 4-His-Fe dioxygenase NOV1 ^17^ (PDB ID: 5J53), which binds molecular oxygen^19^ at its active site, *Map*ADO shows a similar structure. The β-propeller motif is versatile, as seen in lignostilbene dioxygenase from *Pseudomonas brassicacearum* (PDB ID: 5V2D),^20^ with a similar fold (RMSD = 0.85 for 374 Cα atoms).

**Figure 1:**
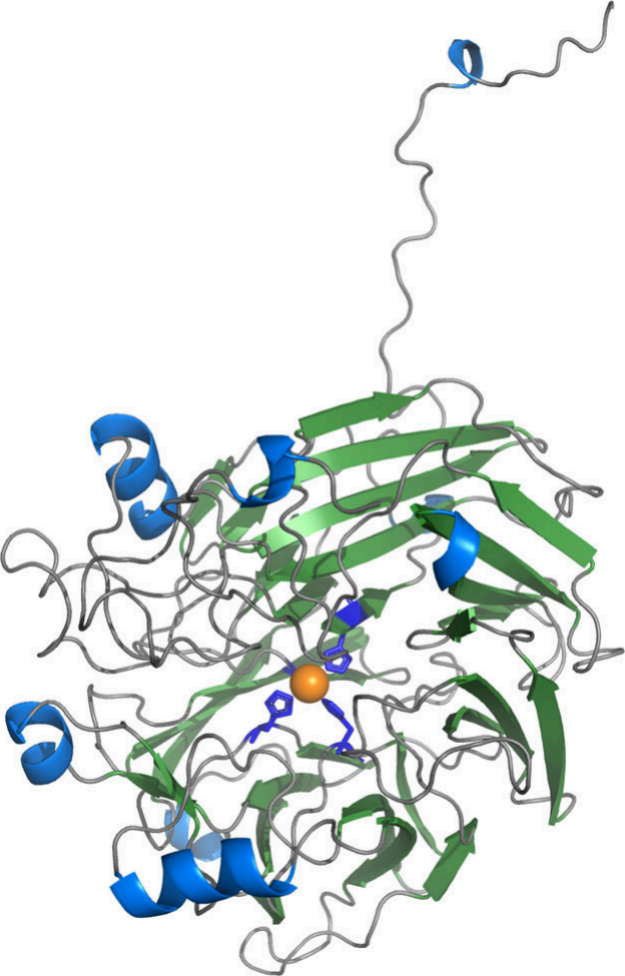
Overview of monomeric *Map*ADO. Secondary structures are color-coded: blue for helices, green for beta sheets, and gray for random coils. Histidines are shown as blue licorices, and the Fe^2+^ ion is shown as an orange sphere.

Although *Map*ADO presents two molecules in the asymmetric unit (SI Figure S10), biophysical analyses suggest it is monomeric in solution: It eluted at 9.6 mL upon size exclusion chromatography on a Superdex 75 10/300 GL column, indicating a protein size of ca. 60 kDa which is consistent with its theoretical molecular weight (62.8 kDa) (SI Figures S11–S12).

*Map*ADO was cocrystallized with excess (40-fold mol) ligands **3** and **1** (products of **3a** and **1a** cleavage, respectively). The ligands are well-described by their respective electron densities (SI Figure S13). Fe^2+^ is coordinated to H200, H251, H316, and H510 and a water molecule. The sixth coordination site is occluded by T156.^21^ In the **1**-bound structure (PDB ID: 9G89), interactions include hydrophobic contacts (I194, F340) and hydrogen bonds with K169, Y136, and E170 via a water bridge (Figure 2A). K169 also stabilizes **1** by interacting with its methoxy oxygen. The **3**-bound structure (PDB ID: 9G8A) shows similar hydrophobic interactions and hydrogen bonds but lacks K169 binding to a methoxy group (Figure 2C). Acetate bound in product-free crystals formed hydrophobic interactions (L41, F340), indicating stable but nonspecific coordination (Figure 2B).

**Figure 2:**
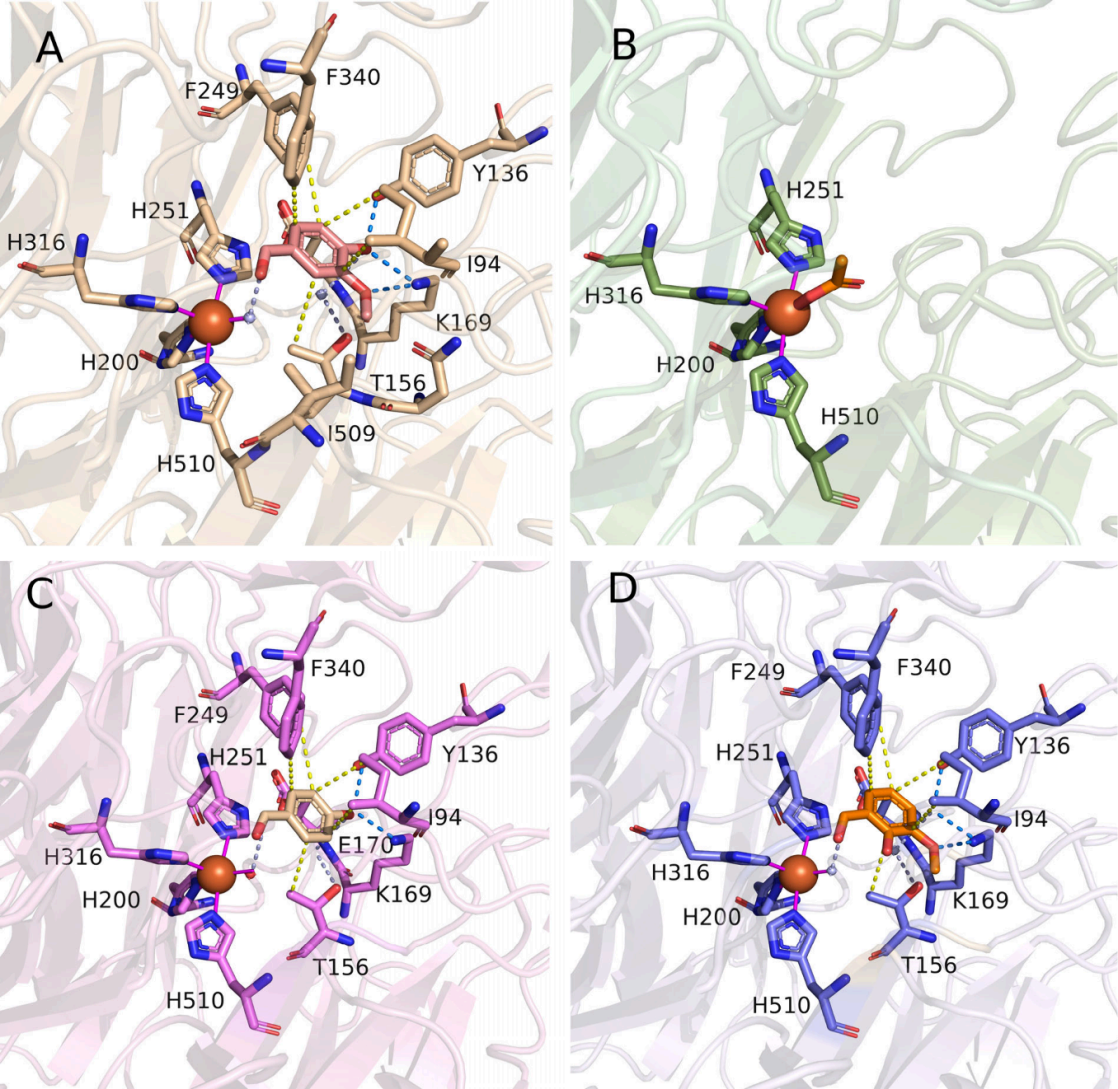
Close up views of ligands bound to *Map*ADO crystals. A: *Map*ADO with vanillin **1**, B: *Map*ADO with acetate, C: *Map*ADO with HBA **3**, D: *Map*ADO with *ortho*-vanillin **5**; *Map*ADO shown as a cartoon with important residues and ligands shown as licorice. Interactions between histidines and Fe^2+^ shown as solid line magenta. Hydrogen bonds shown as blue dashed lines. Hydrophobic interactions shown as yellow dashed lines. Water bridges shown as pale blue dashed lines.

Vanillin (**1**) and HBA (**3**) align well with the *Neurospora crassa* CCO structure in complex with **3a** (PDB: 5U90), where the active Fe^2+^ had been replaced with Co^2+^ (SI Figure S14)^17^ validating *Map*ADO for substrate modeling. While **5a** is not a substrate (as **5** was undetectable in reactions), **5** can bind at the active site. The **5**-bound structure (PDB ID: 9G8F) aligns with **1** and **3** but positions the 2-OH group in a hydrophobic environment (L41, I94, I509), unlike the 4-OH group, which forms polar interactions. This suggests substrate activation relies on polar interactions.

The presence of **5** as a ligand (Figure 2D) suggests it may act as a competitive inhibitor. Adding equimolar **1** reduced **1a** conversion by 60%, whereas **5a** and **5** showed minor inhibitory effects (12% and 25%, respectively, SI Figure S15), pointing at higher affinity for **1** over its *ortho*-isomer.

*Map*ADO’s catalytic pocket lacks a conventional substrate access tunnel.^22^ Instead, it features a confined cavity closed by three Phe side chains, unlike other CCO structures with tunnels leading to the catalytic Fe. Docking simulations revealed a secondary “back pocket” behind the Fe-coordinating histidines, accessible via a tunnel (Figure 3B). A recent study on *T. thermophila* ADO (*Tt*CCO) utilized AlphaFold2 modeling to dock 2-methoxy-4-vinylphenol into this back pocket and the access tunnel of the backside pocket was engineered.^23^ However, this pocket is unlikely to support catalysis because: 1) histidines shield the substrate from Fe; 2) the substrate’s double bond is too distant from Fe; and 3) product-loaded crystal structures and all known ADO structures with ligands confirm ligand binding in the opposite (front) pocket, where substrates are optimally positioned for catalysis (SI Figure S14). **1a** docked into the back pocket (orange sticks) is distant from the dioxygen ligand, confirming this as a nonactive site (Figure 3B). These findings reinforce the front pocket as *Map*ADO’s active site.

**Figure 3:**
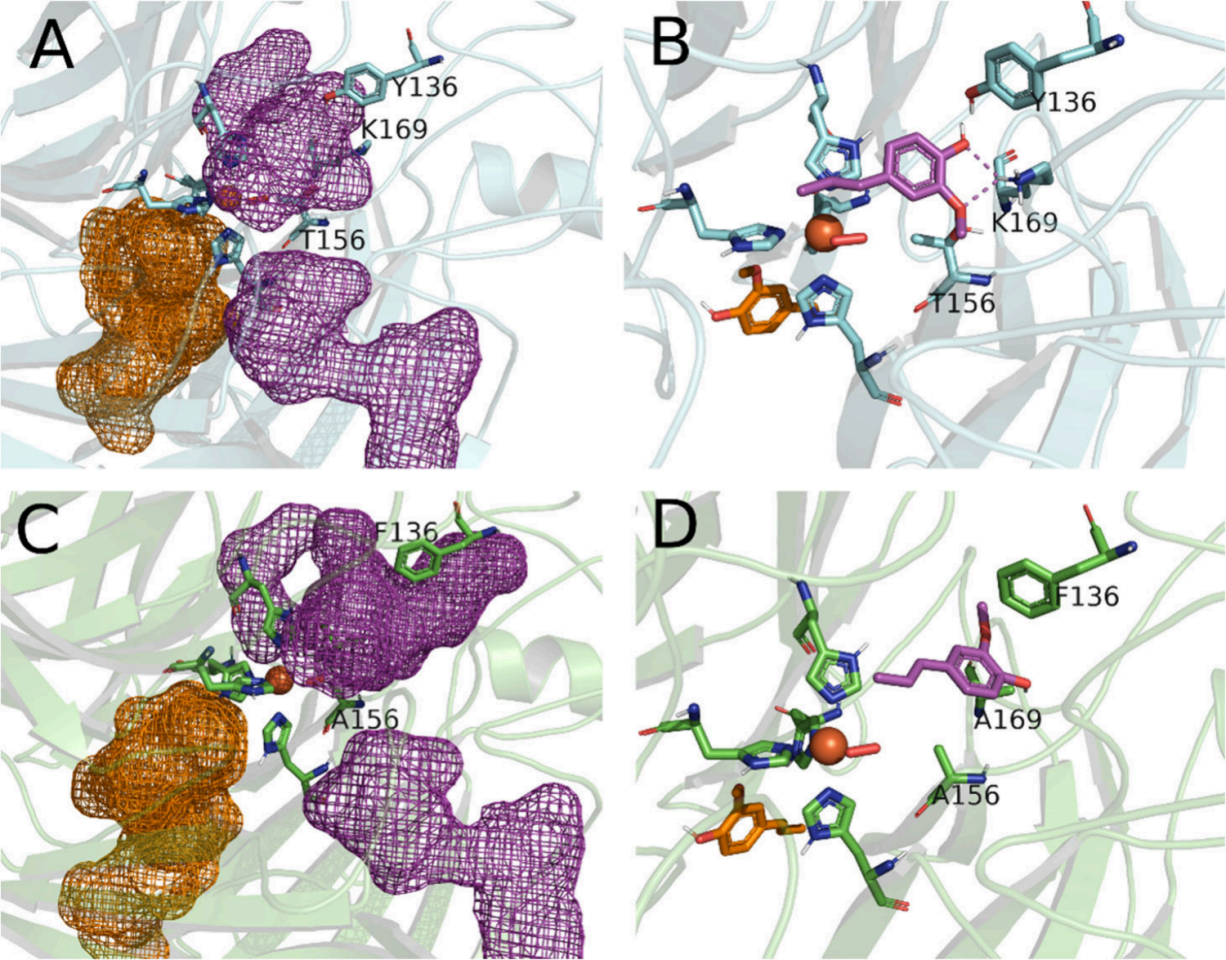
Homology modeling of O_2_ containing active site of *Map*ADO; binding pockets and interactions with **1a**, A: Size and morphology of two binding pockets of *Map*ADO with a larger, surface accessible pocket pointing to the back of the histidine metal coordination sphere (orange mesh) and proximal pocket to O_2_ (magenta mesh), B: Docking of **1a** in the front pocket (magenta sticks) forming hydrogen bonds to K169 (magenta dashed lines). **1a** docked into the back pocket (orange sticks), C: Change in size and morphology of the front pocket of *Map*ADO triple mutant (magenta mesh), D: Docking of **1a** in the front pocket of *Map*ADO triple mutant (magenta sticks) and the back pocket (orange sticks).

Since no oxygen-bound ADO models are available, we used homology modeling based on *Map*ADO crystal structures and a dioxygen-bound NOV1 model (PDB: 5J54). The dioxygen ligand position was transferred to a *Map*ADO model, refined via DFT, and used to analyze active site morphology and function (SI Figure S16–17). Docking simulations^24,25^ confirmed two binding pockets: the surface-accessible back pocket (orange mesh) and a smaller front pocket near the dioxygen ligand (violet mesh), which contains key residues (K169, Y136, T156) forming hydrogen bonds, similar to **3a**-bound NOV1 (Figure 3A), (PDB: 5J54). Enhancement of catalytic activity of *Tt*CCO through back-side tunnel engineering miraculously resulted in five variants—K192N, V310G, A311T, R404N, and D407F—with improved catalytic activity (*k*_cat_/*K*_m_) for 4-vinyl guaiacol.^23^

These mutations corresponding to *Tt*CCO variants were introduced into *Map*ADO (K202N, T317G, A318T, Q394N, N397F, Figure 4) and tested for **1** production from **1a**. We deliberately chose conditions under which *Map*ADO WT would not show full conversion (40 mM **1a**, reaction time 30 min). Q394N and N397F variants showed enhanced production (11.73 mM and 10.52 mM, respectively) compared to WT (8.80 mM), while other variants showed lower activity (SI Figure S18). These variations correlated strongly with the expression levels of the respective variants (SI Figure S19).

**Figure 4:**
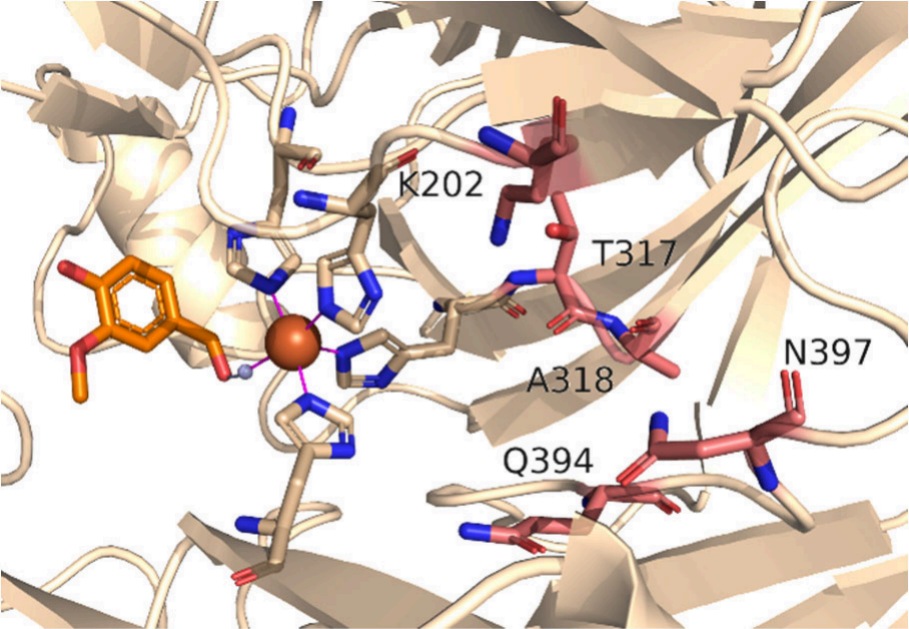
Crystal structure of *Map*ADO with vanillin. Five residues from alignment to *Tt*CCO are shown in pink. The Fe^2+^ center, coordinating histidines and vanillin are shown in brown, beige and orange, respectively.

The role of Y136 and K169 was hypothesized to be substrate activating by hydrogen bond formation with the 4-OH group,^19^ or correct substrate positioning.^26^ Docking simulations showed that **1a**’s hydroxyl and methyl ether groups interact with K169 (Figure 3B), and its double bond is positioned for oxidation (see also SI Figure S20). Experimental analysis of Y136, T156, and K169 (Table 3, SI Table S3) substitutions revealed Y136A strongly reduced activity, while Y136F retained function, hinting at a structural function of this residue. The K169A variant displayed minimal product formation, confirming K169’s critical role. Double and triple variants (Y136F/K169A, T156A/K169A, and Y136F/T156A/K169A, Figure 3C and D) showed activity levels consistent with K169A alone. Computational analyses supported these findings, showing mutations disrupted K169’s hydrogen bonding, enlarged the active pocket and shifted the ligand (Figure 3D) away from the iron center.

**Table 3:** Vanillin Produced by Active Site Mutants^a^

	**1** [mM]	
*Map*ADO variant	1 h	24 h
Wild type	19.73 ± 1.88	19.82 ± 0.07
Y136A	1.00 ± 0.03	2.08 ± 0.05
Y136F	8.94 ± 0.17	13.19 ± 0.12
T156A	19.54 ± 0.83	19.87 ± 0.08
Y136F/T156A	11.84 ± 1.13	14.23 ± 1.73
K169A	0.93 ± 0.00	3.26 ± 0.06
Y136F/K169A	0.36 ± 0.01	0.59 ± 0.05
T156A/K169A	0.62 ± 0.02	1.02 ± 0.03
Y136F/T156A/K169A	0.015 ± 0.003	0.38 ± 0.05

a Conditions: 20 mM isoeugenol, whole cells (OD_600_ 10), in potassium phosphate buffer (10 mM, pH 7.4) with 2 vol% of EtOH at 40°C on a tissue culture rotator; reaction time: 1 and 24 h. Reactions were carried out in technical triplicates. See SI Figure S19 for expression levels.

In conclusion, this study provides an in-depth exploration of the structure and function of a novel oxygenase from *Moesziomyces aphidis* (*Map*ADO) that is outstanding in terms of catalytic activity (*k*_*cat*_ 238 s^–1^, *V*_*max*_ 231 μmol min^–1^ mg^–1^) and strength of iron binding. *Map*ADOs pH range is broad, and it is insensitive to freeze/thaw cycles both in purified form and as *E. coli* resting cell catalyst. These features render *Map*ADO a promising candidate for applications in synthesis.

Detailed comparisons between ligand positions observed in four crystal structures and those predicted in previously published computational models reveal notable substrate positioning and orientation inconsistencies.^23^ These findings underscore the risks of overreliance on computational modeling without experimental validation and highlight the challenges of concluding function solely from structural similarity, even among highly analogous proteins. A diverse and precise data set of experimentally determined structures is provided to achieve reliable predictions. By site-directed mutagenesis we identified K169 as the most important residue for activity, although Y136 and T156 can partly rescue its role in hydrogen-bonding.

Our results emphasize the remarkable potential of *Map*ADO, positioning it as a promising biocatalyst for sustainable vanillin production and contributing to the advancement of green chemistry applications by supporting the development of eco-friendly industrial processes.

## Supplementary Material



















## Notes

Experimental descriptions and additional figures and tables; map density files, PDB files, and PDB validation (PDF)
9G8A (CIF)
9G8A (PDB)
9G8F (CIF)
9G8F (PDB)
9G88 (CIF)
9G88 (PDB)
9G89 (CIF)
9G89 (PDB)

## Abbreviations

ADO: Aromatic Dioxygenase
CCO: carotenoid cleaving oxygenase
HBA: 4-hydroxybenaldehyde
OD: optical density
PDB: Protein Data Bank
