# Supplementary material for: Structural and Functional Characteristics of Potent Dioxygenase from Moesziomyces aphidis
Source: JACS Au. 2025 Jun 12;5(7):3014–20. doi: 10.1021/jacsau.5c00456 (PMC12308385; doi:10.1021/jacsau.5c00456)
Supplement: Supplementary file 1 [file au5c00456_si_001.pdf]

Structural and Functional characteristics of potent dioxygenase from *Moesziomyces aphidis*

Lukas Schober,[†,8] Jacek Plewka,[‡,8] Kanokkan Sriwaiyaphram,[§] Björn Bielec,[#] Astrid Schiefer,[#] Thanyaporn Wongnate,[§] Katarzyna Magiera-Mularz,[‡] Florian Rudroff,[*,#] Margit Winkler[*,†,[illegible]]

[†]Institute of Molecular Biotechnology, Graz University of Technology, NAWI Graz, Petersgasse 14, 8010 Graz, Austria.

[‡]Department of Organic Chemistry, Faculty of Chemistry, Jagiellonian University, Gronostajowa 2, Krakow, Poland

[§]School of Biomolecular Science and Engineering, Vidyasirimedhi Institute of Science and Technology (VISTEC), Wangchan Valley, Rayong, 21210, Thailand

[#]Institute of Applied Synthetic Chemistry, TU Wien, Getreidemarkt 9, 1060 Wien, Austria

[illegible]Austrian Center of Industrial Biotechnology, Krenngasse 37, 8010 Graz, Austria

Table of contents

Supplementary figures 3

Supplementary tables 14

Materials and Methods 16

General 16

Strains and Plasmids 16

Preparation of constructs 16

Preparation of *Map*ADO Variants 20

General flask expression protocol 20

Biotransformation reactions with *Map*ADO 21

Enzyme purification 21

Biophysical Characterization 22

Determination of Crystal Structures 22

Homology modelling of the oxygen containing active site of *Map*ADO 23

Mutation and docking studies 23

Removal of Fe^{2+} ion from purified *Map*ADO 23

Inhibition experiments 24

Spectrophotometric assay for determination of kinetic parameters 24

Determination of kinetic parameters by single endpoint UPLC measurements 24

HPLC-UV analysis 24

References 24

Supplementary figures

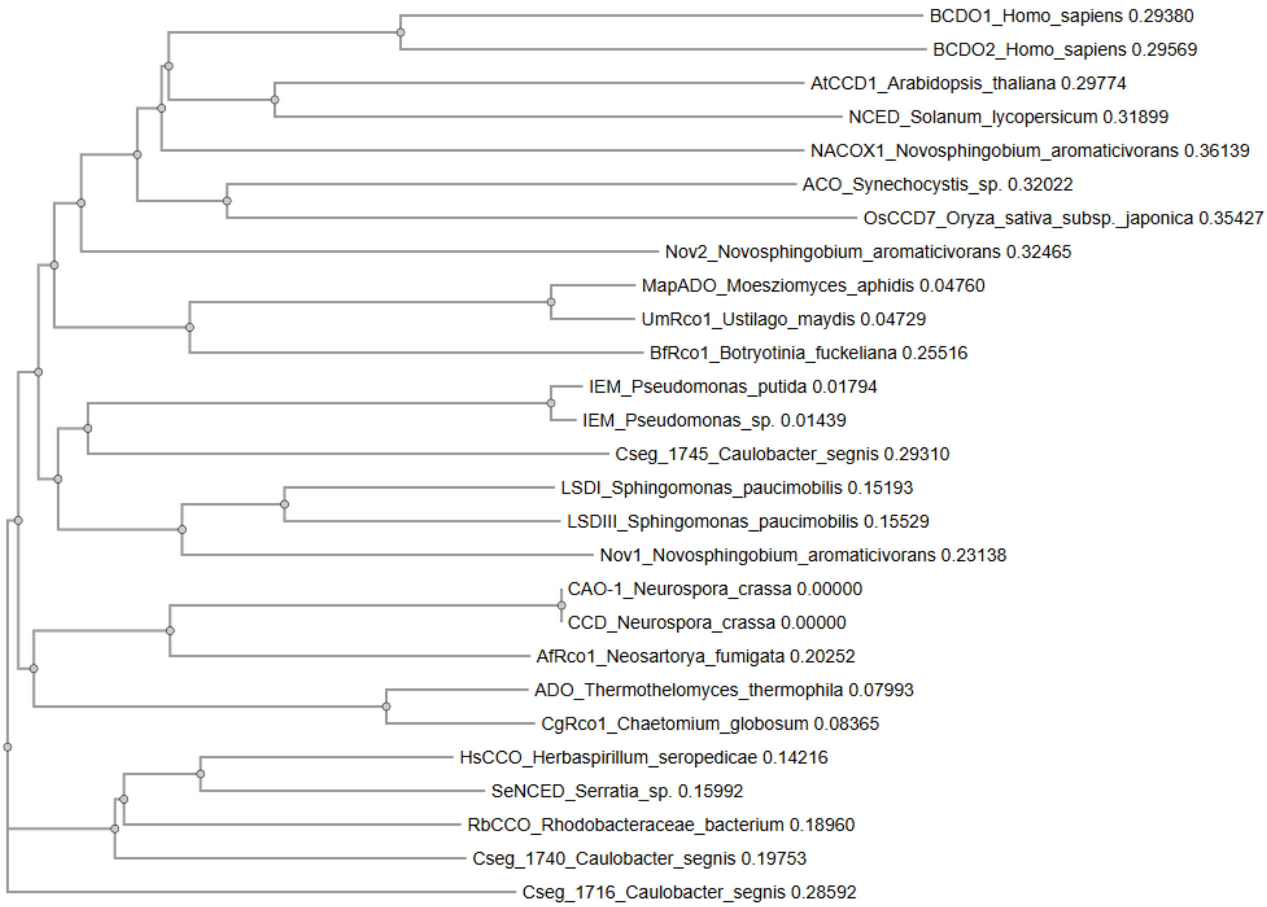

Figure S1: Phylogenetic tree of several carotenoid cleaving oxygenases (CCOs)

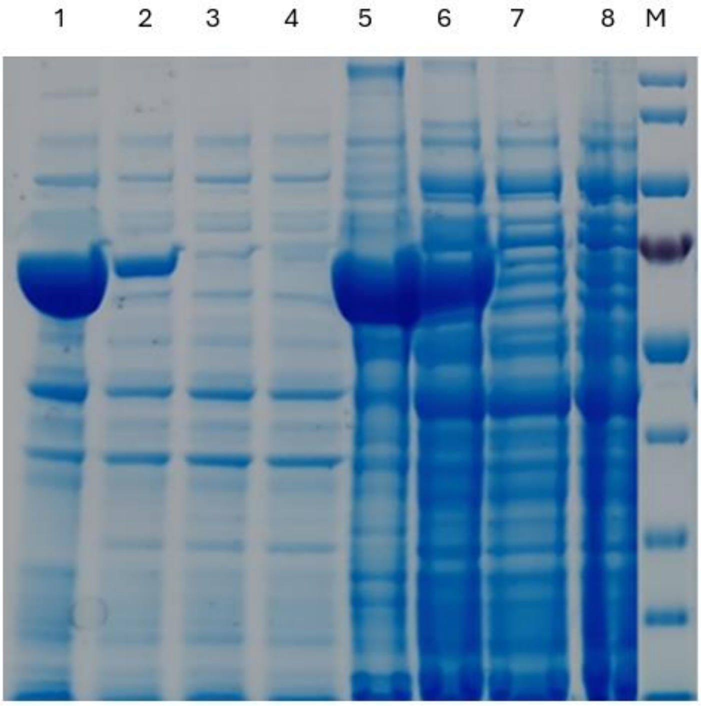

Figure S2: SDS-PAGE with Coomassie blue staining of MapADO with various tags; lane 1, soluble fraction of cell lysate of N-term His-tagged MapADO; lane 2, soluble fraction of cell lysate of N-term StrepII-tagged MapADO; lane 3, soluble fraction of cell lysate of C-term StrepII-tagged MapADO; lane 4, soluble fraction of cell lysate of untagged MapADO; lane 5, insoluble fraction of cell lysate of N-term His-tagged MapADO; lane 6, insoluble fraction of cell lysate of N-term StrepII-tagged MapADO; lane 7, insoluble fraction of cell lysate of C-term StrepII-tagged MapADO; lane 8, insoluble fraction of cell lysate of untagged MapADO; M, protein standard marker. All protein fractions are produced in E. coli BL21 (DE3) induced with 1 mM IPTG and 1 mM $FeCl_2$ added, expression at 20°C/100 rpm for ~ 18 h.

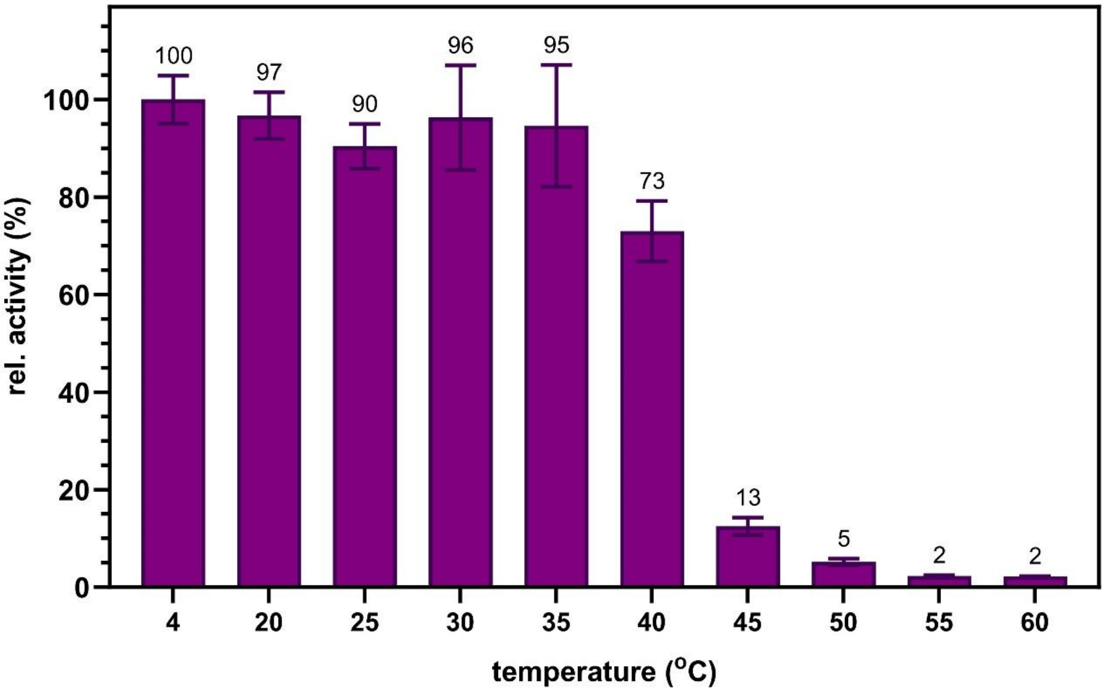

Figure S3: Relative conversions with purified MapADO after 20 min heat treatment at different temperatures: heat treatment at 40°C, 45°C, 50°C, 55°C and 60°C without agitation; 20 mM isoeugenol, purified enzyme (0.7 mg/mL), in potassium phosphate buffer (10 mM, pH=7.4) with 2 vol % of EtOH at 40°C/600 rpm in a thermoshaker; reaction time: 2 h (reactions prepared in technical triplicates)

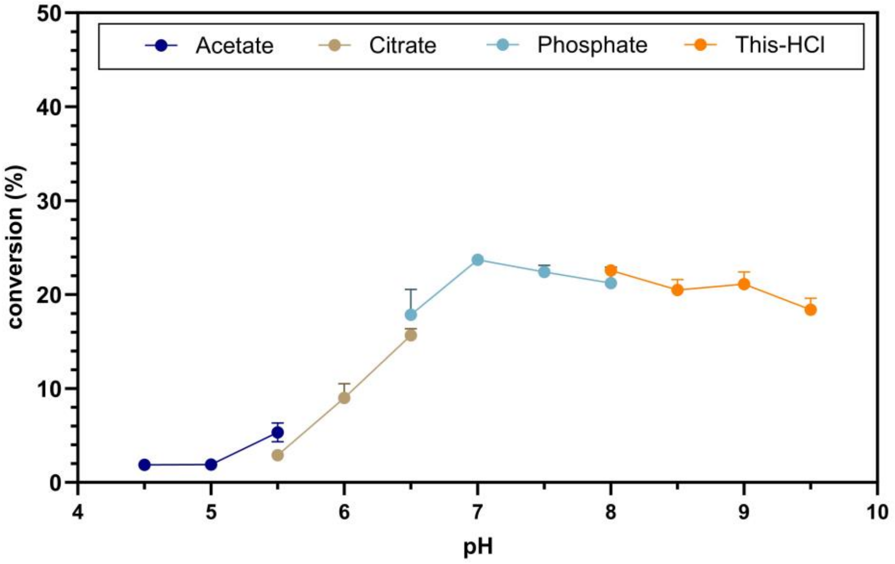

Figure S4: Conversion of isoeugenol to vanillin with purified MapADO (0.2 mg/mL); reaction time 1 h; reaction temperature: 30°C; all reactions were prepared in triplicates

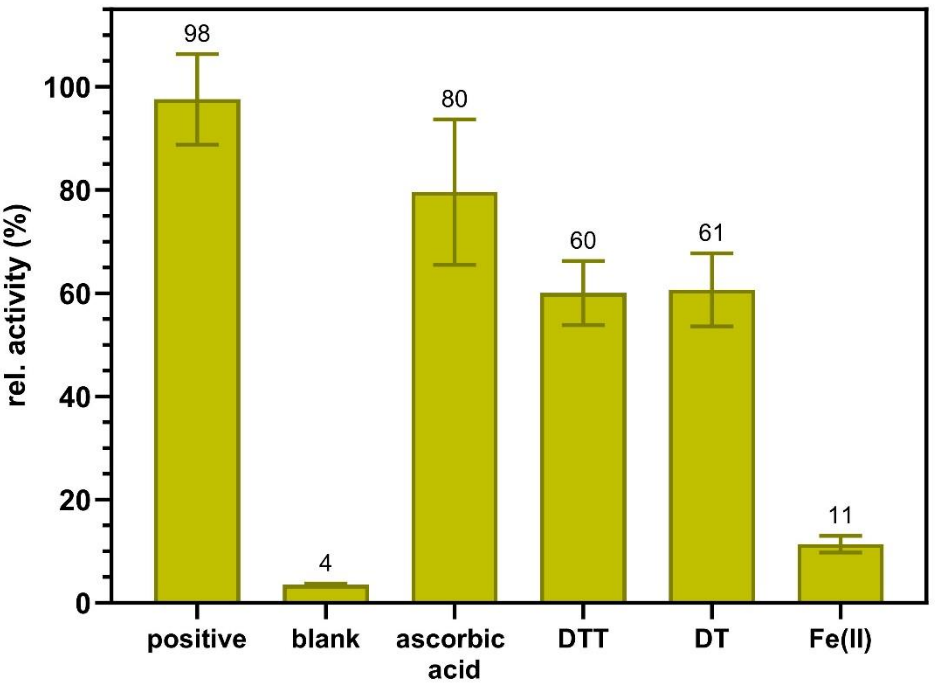

Figure S5: Relative conversions with purified MapADO after addition of 10 mM reducing agent [ascorbic acid, sodium dithionite (DT), dithiothreitol (DTT), $FeCl_2$]: purified enzyme (1 mg/mL), in potassium phosphate buffer (10 mM, pH=7.4) incubated with 10 mM reducing agent for 2 h at 4°C/600rpm in a thermoshaker; then addition of 20 mM isoeugenol with 2 vol % of EtOH; incubation at 40°C/600 rpm in a thermoshaker; reaction time: 2 h (reactions prepared in technical triplicates)

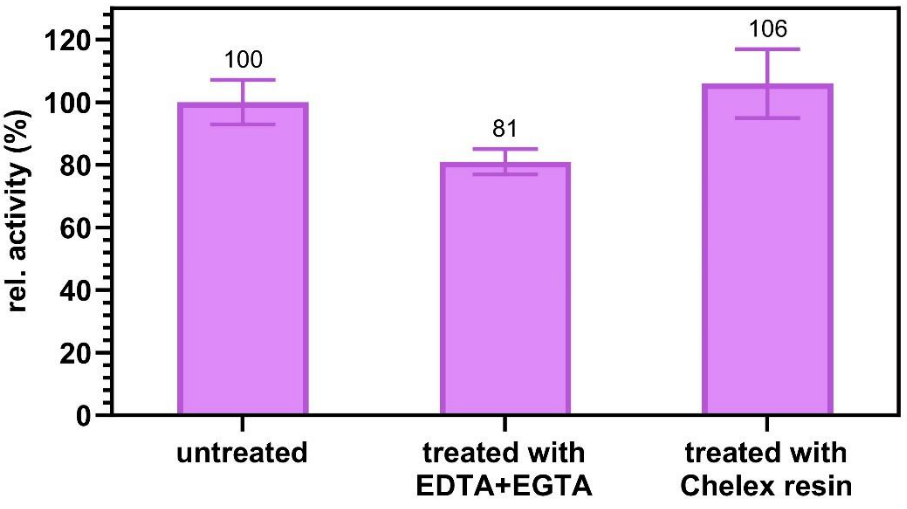

Figure S6: Comparative analysis of the conversion of isoeugenol to vanillin catalyzed by treated and untreated MapADO. Reaction mixture: purified enzyme (0.6 mg/mL) in potassium phosphate buffer (10 mM, pH=7.4), 20 mM isoeugenol with 2 vol % of EtOH at 40°C/400 rpm on a thermoshaker; reaction time: 3 h, reactions prepared in technical triplicates

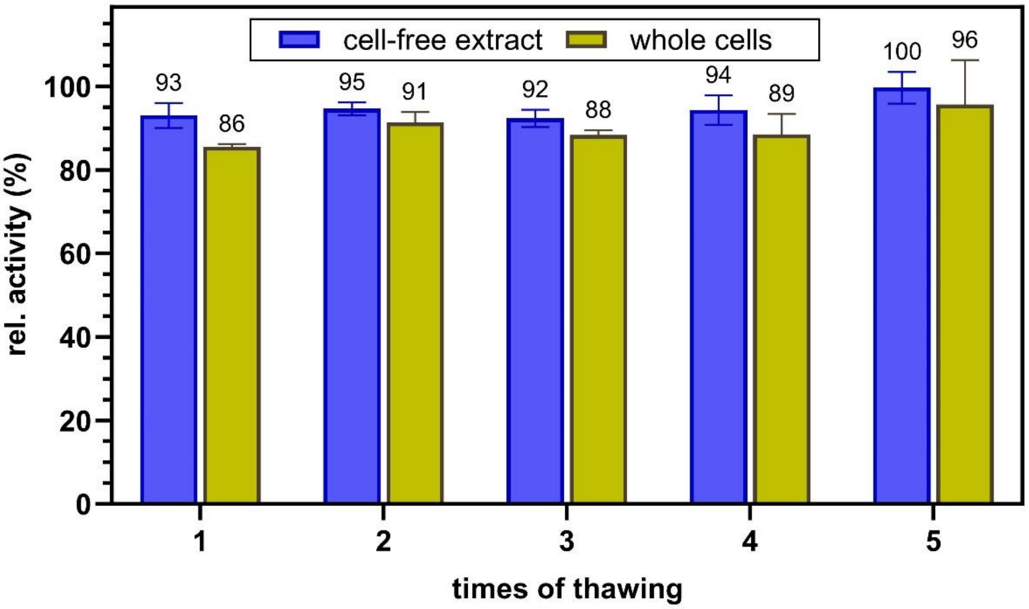

Figure S7: Relative activity after cycles of freezing and thawing; 20 mM isoeugenol, cell-free extract (~40 mg/mL) (blue) or whole cells (OD_{600} 10) (green), in potassium phosphate buffer (10 mM, pH=7.4) with 2 vol % of EtOH at 40°C on a tissue culture rotator; reaction time: 15 min (reactions prepared in technical triplicates)

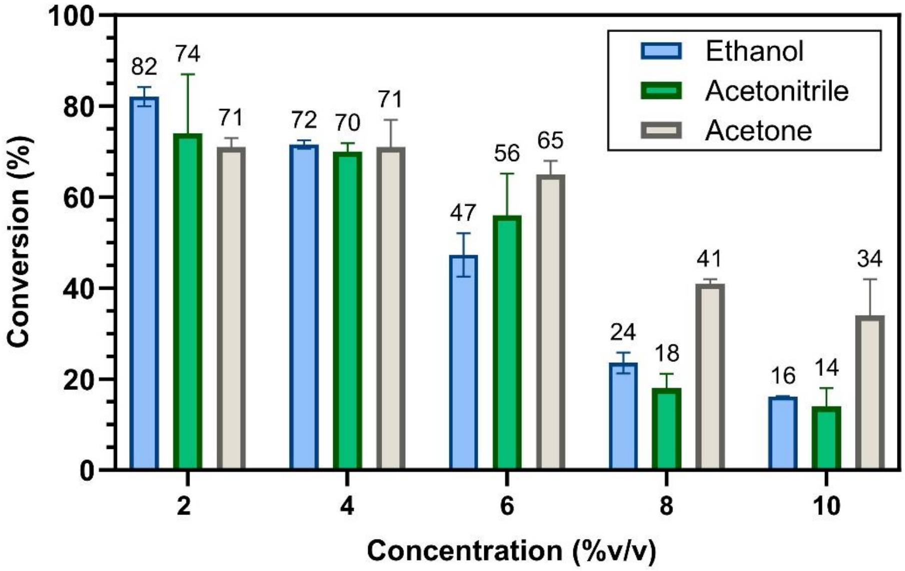

Figure S8: Relative activity. 20 mM isoeugenol, cell-free extract (4.0 mg/mL), in potassium phosphate buffer (10 mM, pH=7.4) with 2 vol % of EtOH at 40°C/600 rpm in a thermoshaker; reaction time: 60 min (reactions prepared in technical triplicates)

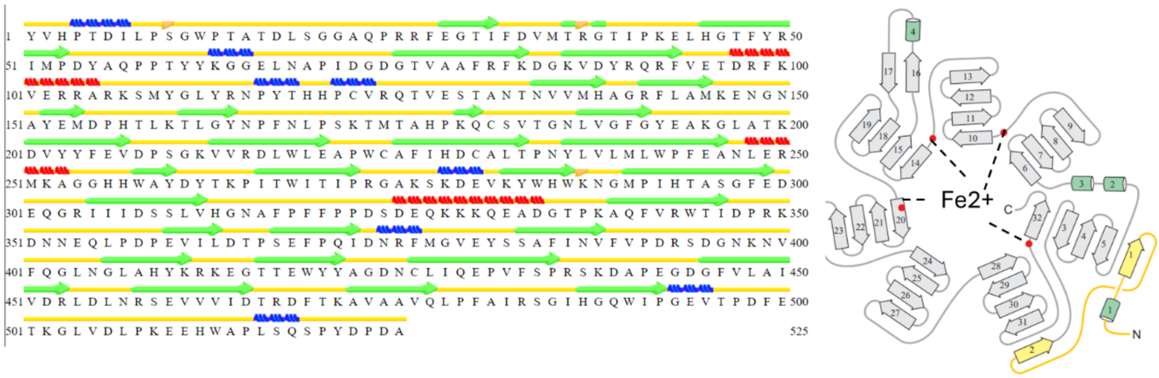

Figure S9: Left: Overview of the secondary structure of MapADO. Helices are shown in blue, β-sheets in green, turns in red, and random coils in yellow. Right: Overview of the secondary structure topology of MapADO, including β-sheet numbering and the positions of histidines (red dots) interacting with the central Fe^{2+} ion.

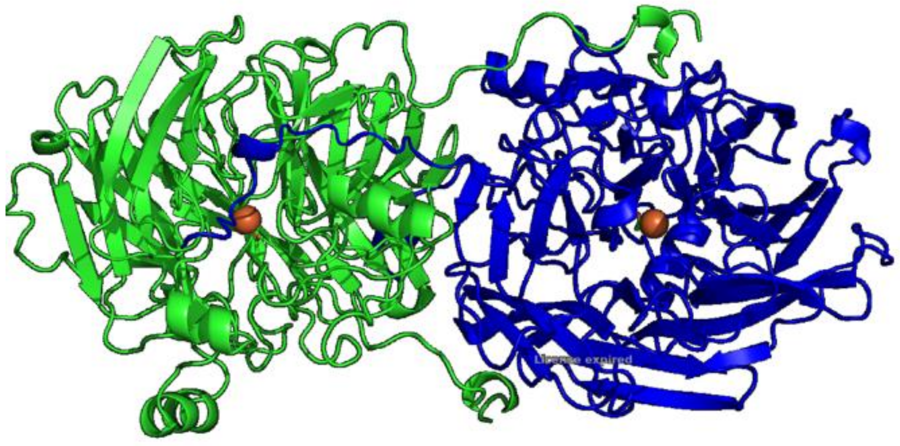

Figure S10: MapADO crystalized as a dimer

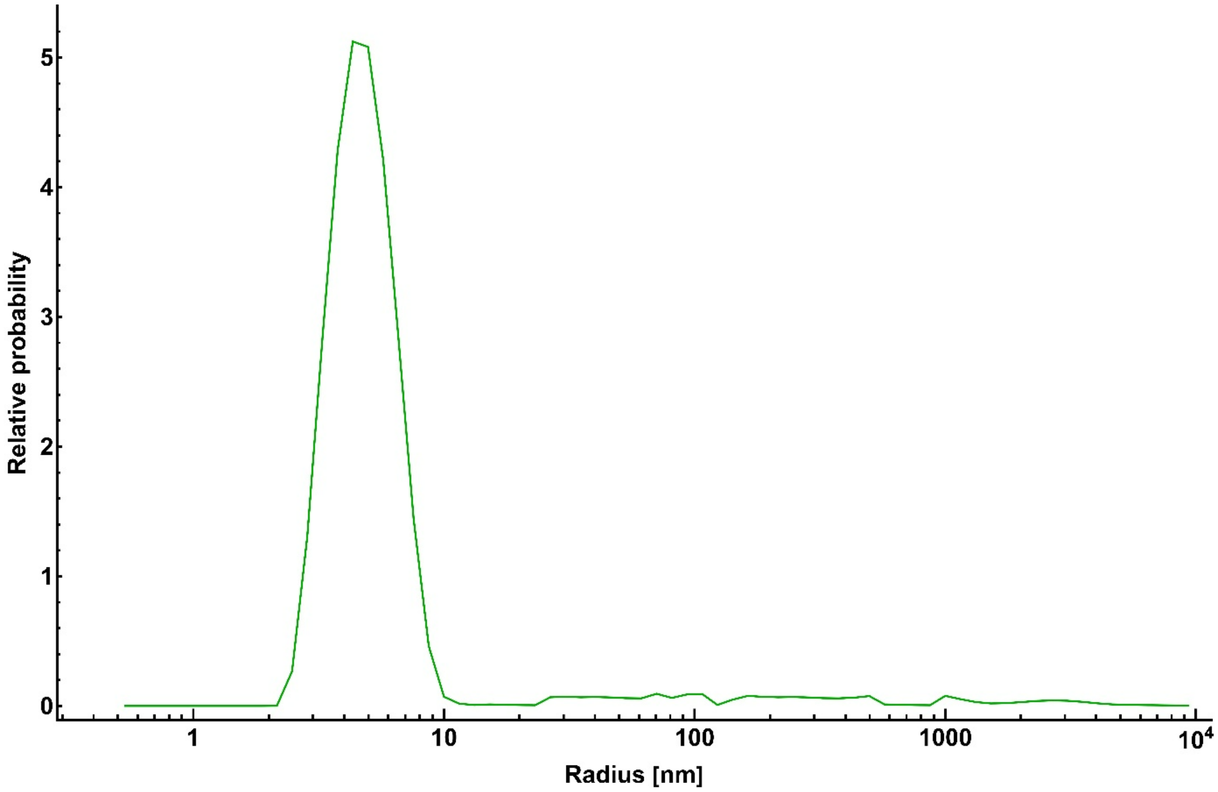

Figure S11: Dynamic light scattering profile of MapADO showing monomeric protein with hydrodynamic radius of 4.3 nm.

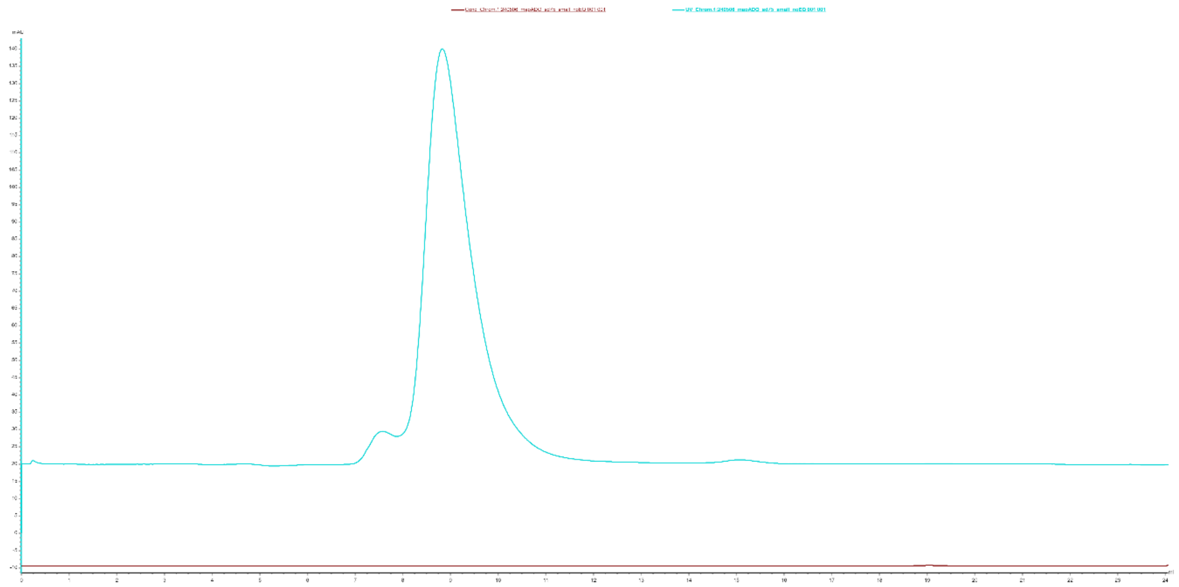

Figure S12: Size Exclusion Chromatography (SEC) of MapADO using a Superdex 75 10/300 GL column (Cytiva) showed a single main peak at an elution volume of 9.6 mL. This elution volume corresponds to an approximate molecular weight of ~60 kDa, indicating that MapADO is monomeric in solution. This is consistent with the calculated molecular weight of 62.8 kDa based on its amino acid sequence.

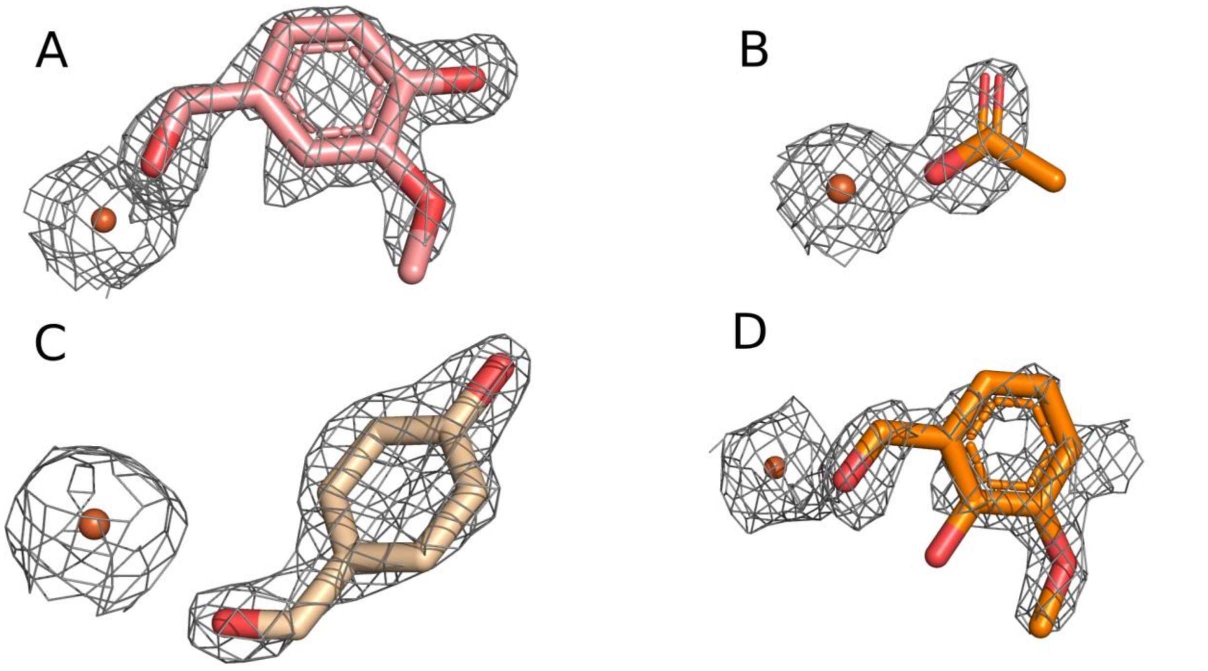

Figure S13 Omit maps Fo − Fc for A: MapADO with vanillin and Fe^{2+} 1, B: MapADO with acetate and Fe^{2+}, C: MapADO with HBA and Fe^{2+} 3, D: MapADO with ortho-vanillin and Fe^{2+}

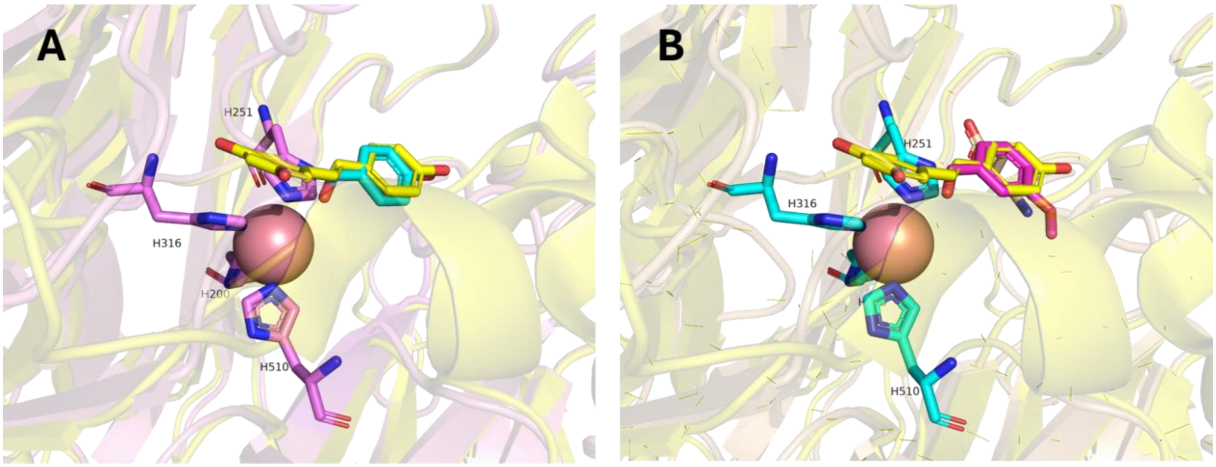

Figure S14 A: Overlay of MapADO structure (pink) with HBA (blue licorice with oxygen atoms red) and CAO1 from PDB ID: 5U90 (yellow) containing resveratrol (yellow licorice with oxygen atoms in red). B: Overlay of MapADO structure (wheat) with vanillin (magenta with oxygen atoms red) and CAO1from PDB ID: 5U90 (orange) containing resveratrol (yellow licorice with oxygen atoms in red).

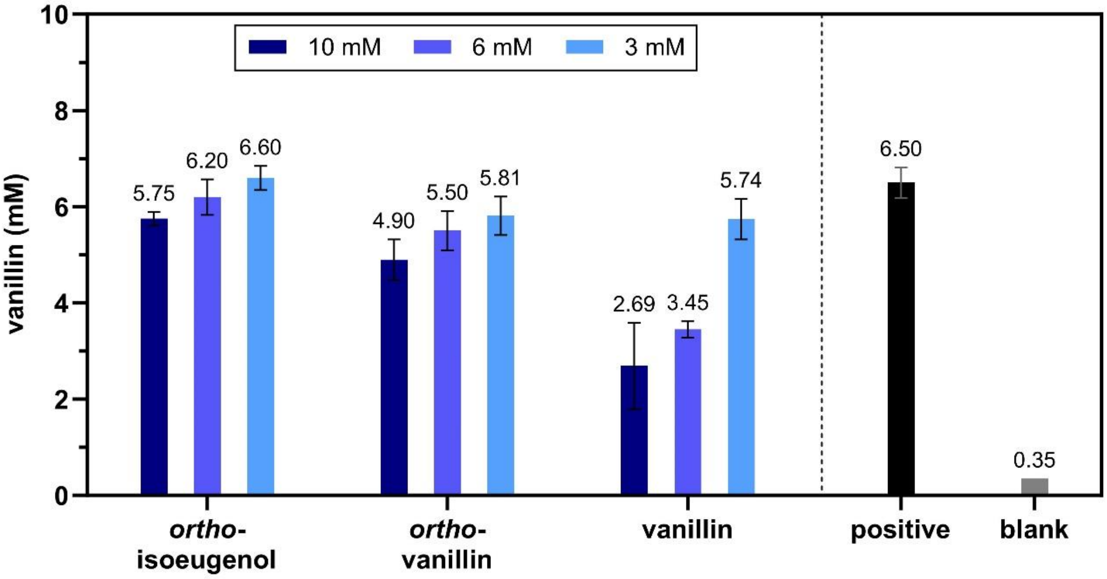

Figure S15: Vanillin formation after adding potential inhibitors. 10 mM isoeugenol, 10 mM, 6 mM or 3 mM inhibitor (ortho-isoeugenol, ortho-vanillin, vanillin), purified enzyme (~0.8 mg/mL) in potassium phosphate buffer (10 mM, pH=7.4) with 2 vol % of EtOH at 30°C/600 rpm in a thermoshaker; reaction time: 90 min, added initial concentration vanillin was subtracted (reactions prepared in technical triplicates)

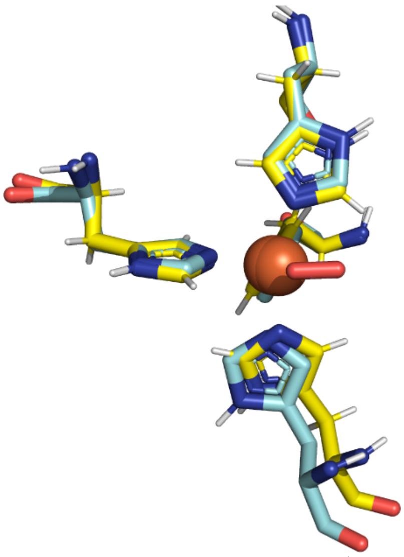

Figure S16: Alignment of the metal binding sphere of our MapADO model (cyan sticks) with removed acetate and water ligand and the NOV1 model (PDB 5j54, yellow sticks) binding dioxygen as ligand in the equatorial plane.

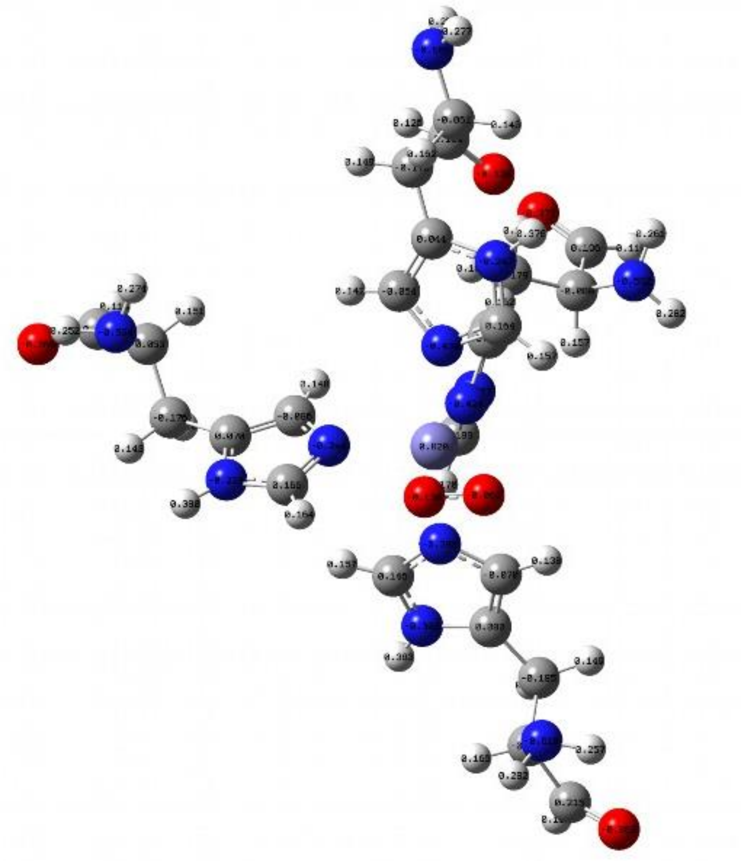

Figure S17: Calculation of the electron densities of the extracted iron binding sphere of MapADO by DFT calculations using the B3LYP functional and the def2tzvp basis set for iron, 6-311G(d,p) for carbon and hydrogens and 6-311+G(d,p) basis set for nitrogen and oxygen

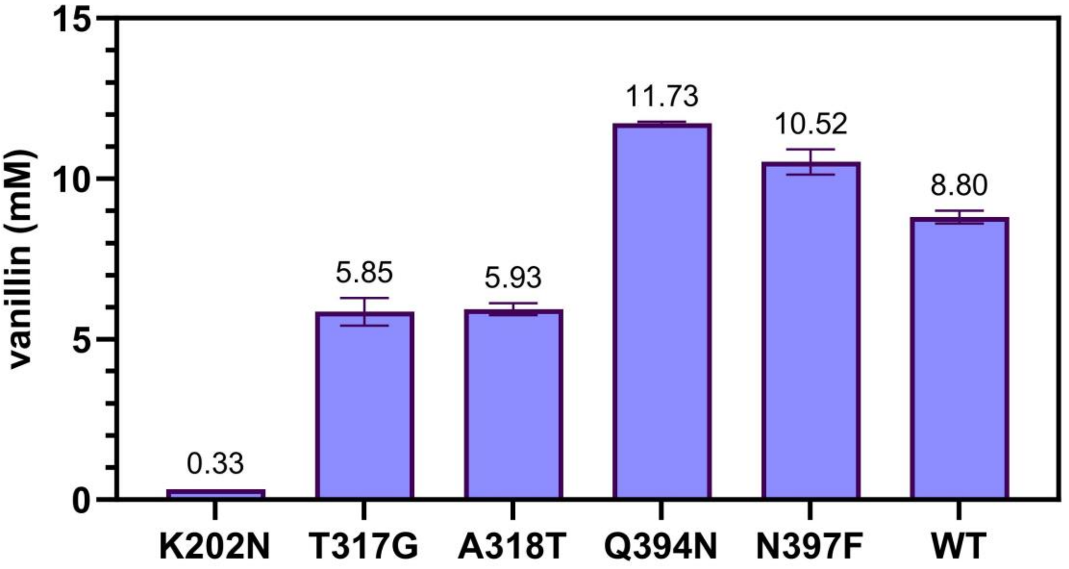

Figure S18: Production of vanillin catalyzed by MapADO variants: whole cells (OD_{600} 6) in potassium phosphate buffer (10 mM, pH=7.4), 40 mM isoeugenol with 2 vol % of EtOH at 40°C/600 rpm; reaction time: 30 min (reactions prepared in technical triplicates)

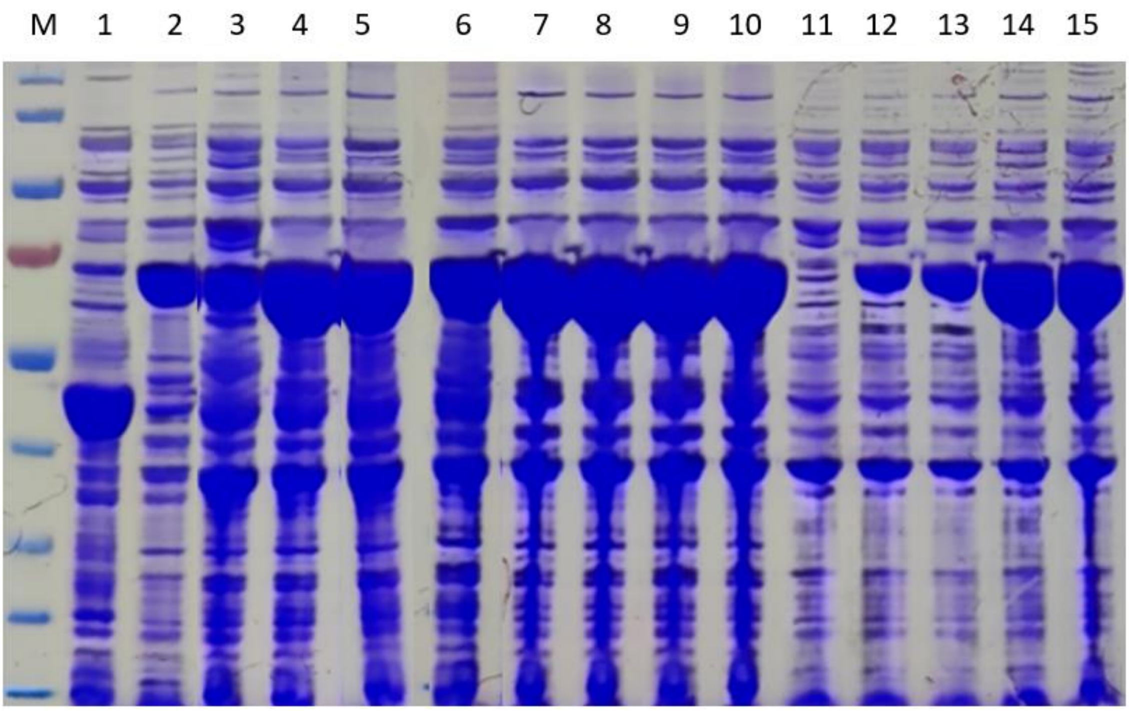

Figure S19: SDS-PAGE with Coomassie blue staining of the soluble supernatant fraction from cell lysate of rational-designed variants of MapADO, protein standard marker; lane 1, EVC (pMS470-D8); lane 2, MapADO wild type; lane 3, MapADO Y136A; lane 4, MapADO Y136F; lane 5, MapADO T156A; lane 6, MapADO Y136F/T165A; lane 7, MapADO K169A; lane 8, MapADO Y136F/K169A; lane 9, MapADO T156A/K169A; lane 10, MapADO Y136A/T156A/K169A; lane 11, MapADO K202N; lane 12, MapADO T317G; lane 13, MapADO A319T; lane 14, Q394N, lane 15, N397F. All MapADO mutants are expressed from a pMS470 vector with an N-terminal His tag; All protein fractions are produced in E. coli BL21 (DE3) induced with 1 mM IPTG and 1 mM $FeCl_2$ added, expression at 20°C/100 rpm for ~ 18 h.

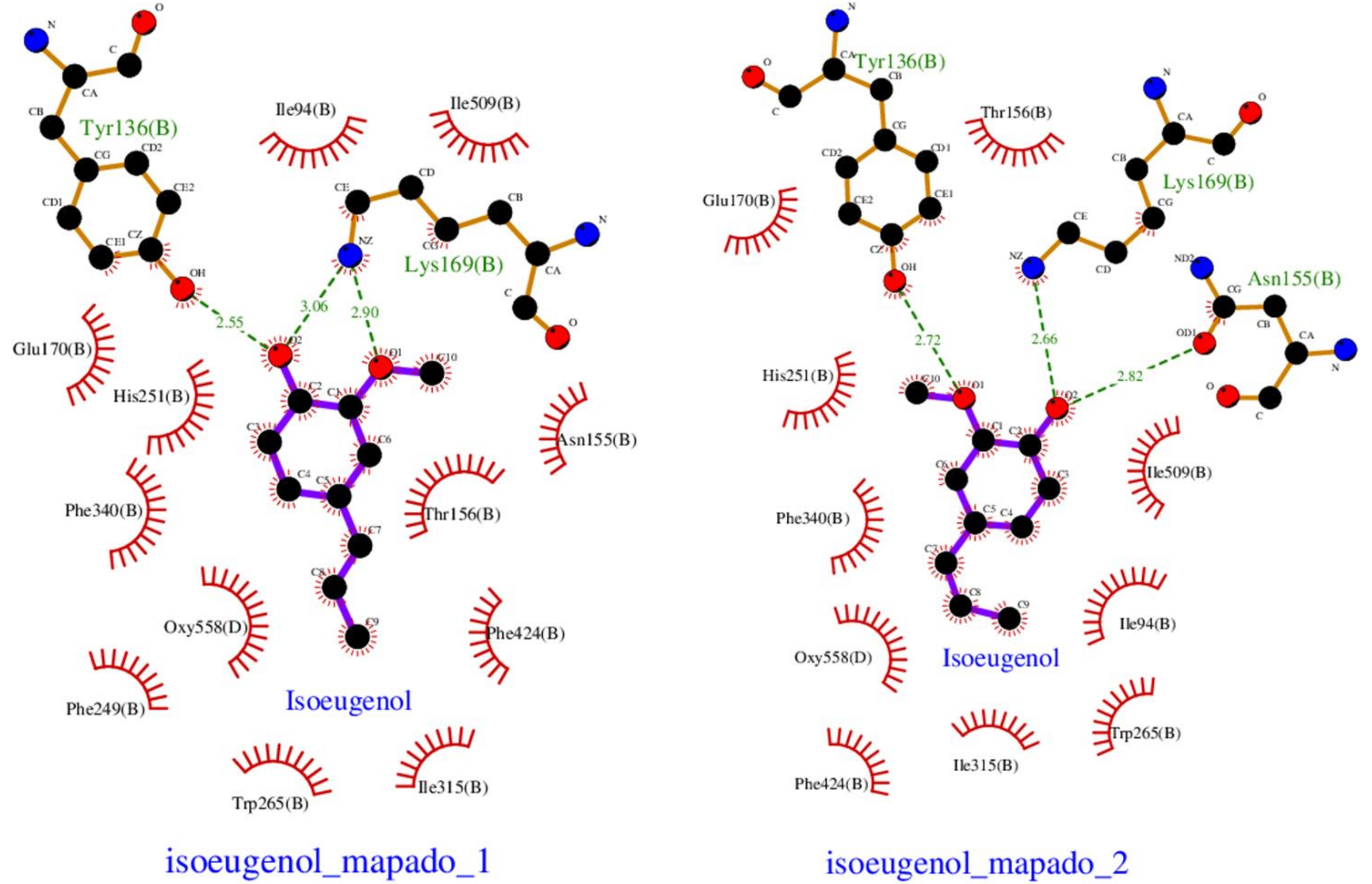

Figure S20: 2D representation of the predominant interactions of isoeugenol with the active site of MapADO. There were only two configurations of isoeugenol inside the active pocket of MapADO.

Supplementary tables

Table S1*: Comparison of kinetic data of selected enzymes with isoeugenol. Note, literature values are based on single end-point measurements. The values determined in this work are based on single endpoint UPLC measurements under conditions described in Ref2*

Entry	enzyme	V_{max} [µmol*min^{-1}*mg^{-1}]	k_{cat} [s^{-1}]	Reference
1	IEM	4.2	3.8	[1]
2	ADO / *Tt*CCO	60	67	[2]
3	*Map*ADO	635	654	this work

Table S2*: Data collection and refinement statistics (molecular replacement).*

PDB ID:	9G8F	9G8A	9G89	9G88
ligand:	ortho vanillin	p-hydroxy benzaldehyde	vanillin	acetate
Wavelength (Å)	1.03324	0.87313	1.03324	1.03322
Space group	P 1 21 1	P 1 21 1	P 1 21 1	P 1 21 1
Cell dimensions				
a, b, c (Å)	71.88 84.94 107.02	71.836 83.808 98.117	71.68 84.676 106.571	72.155 85.957 93.87
α, β, γ (o)	90.00 108.40 90.00	90.0 104.498 90.0	90.0 108.349 90.0	90.0 100.336 90.0
Resolution range (Å)	48.76 - 1.84	69.55 - 2.00	48.56 - 1.67	46.17 - 1.4
Rmerge	0.15 (0.618)	0.111 (0.452)	0.087 (1.473)	0.054 (1.005)
Rpim	0.099 (0.394)	0.110 (0.443)	0.054 (0.911)	0.048 (0.851)
I/σI	9.5 (4.0)	4.5 (1.5)	12.6 (1.2)	9.5 (1.0)
Completeness (%)	96.8	97.1	98.4	97.7
Redundancy	6.5	3.1	7	3.8
Total reflections	658314 (20319)	201331 (15256)	958554 (47539)	828984 (27125)
CC1/2	0.99	0.731	0.528	0.432
Refinement statistics				
Unique reflections	101754 (3118)	67761 (4990)	137992 (6796)	216121 (8729)
Rwork/Rfree	0.2/0.26	0.18/0.22	0.16/0.19	0.17/0.19
Wilson B-factor	16.1	22	21	17.5
Ramachandran favoured (%)	96.8	96.4	96.9	97.5
Ramachandran allowed (%)	3.1	3.6	3.1	2.4
Ramachandran outliers (%)	0.2	0	0	0.1

**High resolution shell in parentheses.*

Table S3. *Vanillin produced by active site mutants with increased cell density compared to Table 3 in the main manuscript. Conditions: 20 mM isoeugenol, whole cells (OD_{600} 60), in potassium phosphate buffer (10 mM, pH=7.4) with 2 vol % of EtOH at 40°C on a tissue culture rotator; reaction time: 1 h and 24 h. Reactions were carried out in technical triplicates. See Fig S18 for expression levels.*

*Map*ADO variant	OD_{600}: 10		OD_{600}: 60	
	**1** [mM] / 1 h	**1** [mM] / 24 h	**1** [mM] / 1 h	**1** [mM] / 24 h
Wild type	19.73±1.88	19.82±0.07	Full conversion	Full conversion
K169A	0.93±0.00	3.26±0.06	2.46±0.05	13.17±1.05
Y136F/K169A	0.36±0.01	0.59±0.05	0.84±0.06	2.46±0.51
T156A/K169A	0.62±0.02	1.02±0.03	3.67±0.13	5.16±0.35
Y136F/T156A/K169A	0.015±0.00	0.38±0.05	0.12±0.02	0.23±0.05

Materials and Methods

General

Standard reagents were obtained from VWR, Sigma-Aldrich (Vienna, Austria) or Roth GmbH & Co. KG (Karlsruhe, Germany). Restriction enzymes were obtained from Thermo Scientific (St. Leon Rot, Germany).

Strains and Plasmids

Escherichia coli Top 10F' or *E. coli* Top 10 was used for molecular cloning and plasmid propagation. *E. coli* BL21 (DE3) was used for the expression of recombinant proteins. A codon-optimized sequence of hypothetical protein PaG_05861 from *Moesziomyces aphidis* (GenBank Accession No: ETS60306.1), herein referred to as *Map*ADO, was cloned into the vector pET-28a and expressed in *E. coli*. *Map*ADO was equipped with affinity tags for purification, and variants were prepared by site directed mutagenesis in pMS-470 vector. *Map*ADO and its mutants were expressed in *E. coli* BL21 using LB medium, with gene expression induced at 20°C by addition of 1 mM of isopropyl β-D-1-thiogalactopyranoside (IPTG) and supplementation with 1 mM of $FeCl_2$.
Bacterial strains and plasmids used are listed in **Table S4** and **Table S5**, respectively.

Table S4: *Used bacterial strains*

Strain	Genotype	Purpose
E. coli BL21 (DE3)	F^- *ompT hsd*S_B (r_B^-, m_B^-) *gal dcm rne*131 (DE3)	Protein expression
E. coli TOP 10 F'	F' [*lac*I^q, *Tn*10(Tet^R)] *mcr*A Δ(*mrr-hsd*RMS-*mcr*BC) φ80*lac*ZΔM15 Δ*lac*X74 *rec*A1 *ara*D139 Δ(*ara-leu*)7697 *gal*U *gal*K *rps*L(Str^R) *end*A1 *nup*G	Plasmid propagation
E. coli TOP 10	F^- *mcr*A Δ(*mrr-hsd*RMS-*mcr*BC) φ80*lac*ZΔM15 Δ*lac*X74 *rec*A1 *ara*D139 Δ(*ara-leu*)7697 *gal*U *gal*K λ^- *rps*L(Str^R) *end*A1 *nup*G	Plasmid propagation

Table S5: *Used plasmids and vectors*

Plasmid	resistance	Usage
pET-21a(+)	Ampicillin	Vector of origin of ADO (*Thermothelomyces thermophilus*)
pET-28a	Kanamycin	Cloning in *E. coli* TOP 10 F', protein expression in *E. coli* BL21 (DE3)
pMS470	Ampicillin	Cloning in *E. coli* TOP 10 F', protein expression in *E. coli* BL21 (DE3)

Preparation of constructs

Table S6: *List of constructs*

#	Name	Tag	Organism of origin	Accession No	Ref.
1	pET-28a-His-MapADO	N-terminal His tag	*Moesziomyces aphidis*	ETS60306.1	This study
2	pET-28a-MapADO	-	*Moesziomyces aphidis*	ETS60306.1	This study
3	pET-28a-StrepII-MapADO	N-terminal Strep II tag	*Moesziomyces aphidis*	ETS60306.1	This study

4	pET-28a-MapADO-StrepII	C-terminal Strep II tag	*Moesziomyces aphidis*	ETS60306.1	This study

Materials from New England Biolabs were used for PCR reactions. Primers were ordered from IDT. PCR reaction fragments were purified using the Wizard® SV Gel and PCR-Clean-Up System kit. Assembled circular vectors were desalted and used for transformation of electrocompetent *E. coli* Top 10F'. Positive clones were selected on LB-Amp or LB-Kan agar, and plasmids were isolated using the Wizard® SV Miniprep DNA Purification System. The sequences were verified through Sanger Sequencing service (Microsynth).

The inserts containing the coding sequence of the *Map*ADO wild-type was ordered as codon-optimized synthetic genes at Twist Biosciences. The DNA sequences were amplified with PCR using Q5 polymerase (primers see **Table S9**) and the DNA fragments were assembled via Gibson assembly after purification with the pET-28a vector that was digested with *Xba*I and *Hind*III before.

The untagged pET-28a-MapADO and both N and C terminal Strep II tagged pET-28a-MapADOs were prepared from pET-28a-His-MapADO by PCR amplification with overhanging primers producing 2 different fragments, one in front of the coding sequence for the *Map*ADO containing the Ribosome-binding site, the lac operator and the T7 promoter (insert 1) and one with the coding sequence (insert 2). Two different fragments of 1 were generated via PCR (**Table S9**): 1a with no tag at the N-terminus of the enzyme and 1b with an N-terminal Strep II tag. Three different inserts 2 were generated: 2a with no tag, 2b with N-terminal Strep II tag and 2c with C-terminal Strep. The complete plasmids are prepared with a Gibson assembly of insert 1 (1a and 1b), interest 2 (2a, 2b and 2c) and the pET-28a backbone that was before digested with *Sph*I and *Hind*III. The combinations of inserts were: 1a and 2a, 1b and 2b and 1a and 2c.

Table S7*: List of mutant variants of MapADO*

#	mutations	vectors
5	Y136A	pMS470-His-MapADO Y136A
6	Y136F	pMS470-His-MapADO Y136F
7	T156A	pMS470-His-MapADO T156A
8	K169A	pMS470-His-MapADO K169A
9	Y136F/T156A	pMS470-His-MapADO Y136F/T156A
10	Y136F/K169A	pMS470-His-MapADO Y136F/K169A
11	T156A/K169A	pMS470-His-MapADO T156A/K169A
12	Y136F/T156A/K169A	pMS470-His-MapADO Y136F/T156A/K169A

Based on crystal structure of ADO from *Moesziomyces aphidis* (*Map*ADO) we produced a total of 4 single-point mutations and 4 multi-point mutations in the pMS470 vector (**Table S7**).

For the preparation of these variants, gene fragments were ordered from Twist Biosciences. Fragments for the preparation of the mutants are all listed in **Table S8**. MUT Y136F/K169A (10), MUT T156A/K169A (11) and MUT Y136F/T156A/K169A (12) were prepared using a combination of the backbones of mutant MUT Y136F (6), MUT T156A (7) and MUT Y136F/T156A (9) and the fragment K169A (F8) (primers see **Table S9**).

Table S8*: List of synthetic gene fragments*

#	Length [bp]	mutations	sequence
F5	321	Y136A	CCCACCGCTACTGACTTATCCGGCGGAGCACAGCCACGTCGTTTTG<br>AGGGTACGATTTTTGACGTGATGACCCGTGGCACAATTCCGAAGGA<br>GCTTCACGGAACTTTTTACCGTATTATGCCTGACTATGCACAGCCAC<br>CTACTTATTACAAGGGAGGAGAACTGAATGCTCCAATTGATGGAGAC<br>GGCACTGTGGCCGCATTTCGCTTCAAGGATGGGAAGGTAGACTACC<br>GTCAACGCTTCGTGGAAACGGATCGTTTTAAGGTCGAGCGTCGCGC<br>ACGTAAATCTATGTACGGTCTGGCCCGCAACCCGTACACGCAC
F6	321	Y136F	CCCACCGCTACTGACTTATCCGGCGGAGCACAGCCACGTCGTTTTG<br>AGGGTACGATTTTTGACGTGATGACCCGTGGCACAATTCCGAAGGA<br>GCTTCACGGAACTTTTTACCGTATTATGCCTGACTATGCACAGCCAC<br>CTACTTATTACAAGGGAGGAGAACTGAATGCTCCAATTGATGGAGAC<br>GGCACTGTGGCCGCATTTCGCTTCAAGGATGGGAAGGTAGACTACC<br>GTCAACGCTTCGTGGAAACGGATCGTTTTAAGGTCGAGCGTCGCGC<br>ACGTAAATCTATGTACGGTCTGTTCCGCAACCCGTACACGCAC
F7	382	T156A	CCCACCGCTACTGACTTATCCGGCGGAGCACAGCCACGTCGTTTTG<br>AGGGTACGATTTTTGACGTGATGACCCGTGGCACAATTCCGAAGGA<br>GCTTCACGGAACTTTTTACCGTATTATGCCTGACTATGCACAGCCAC<br>CTACTTATTACAAGGGAGGAGAACTGAATGCTCCAATTGATGGAGAC<br>GGCACTGTGGCCGCATTTCGCTTCAAGGATGGGAAGGTAGACTACC<br>GTCAACGCTTCGTGGAAACGGATCGTTTTAAGGTCGAGCGTCGCGC<br>ACGTAAATCTATGTACGGTCTGTACCGCAACCCGTACACGCACCAC<br>CCATGCGTACGCCAAACCGTAGAATCGACTGCCAACGCTAACGTCG<br>TCATGCACGCAG
F8	303	K169A	GTCGTCATGCACGCAGGCCGTTTTCTTGCCATGGCCGAAAATGGCA<br>ACGCATACGAAATGGACCCGCACACTCTGAAGACCCTTGGTTATAA<br>CCCGTTCAATTTGCCATCTAAGACTATGACGGCACACCCTAAGCAG<br>TGCTCGGTTACCGGCAATTTGGTGGGCTTCGGATACGAGGCGAAGG<br>GGTTGGCAACCAAAGACGTGTATTATTTTGAGGTGGACCCCTCAGGA<br>AAGGTAGTGCGCGACTTGTGGTTAGAAGCCCCCTGGTGCGCGTTCA<br>TTCACGATTGCGCTCTTACGCCAAAC
F9	382	Y136F/T156A	CCCACCGCTACTGACTTATCCGGCGGAGCACAGCCACGTCGTTTTG<br>AGGGTACGATTTTTGACGTGATGACCCGTGGCACAATTCCGAAGGA<br>GCTTCACGGAACTTTTTACCGTATTATGCCTGACTATGCACAGCCAC<br>CTACTTATTACAAGGGAGGAGAACTGAATGCTCCAATTGATGGAGAC<br>GGCACTGTGGCCGCATTTCGCTTCAAGGATGGGAAGGTAGACTACC<br>GTCAACGCTTCGTGGAAACGGATCGTTTTAAGGTCGAGCGTCGCGC<br>ACGTAAATCTATGTACGGTCTGTTCCGCAACCCGTACACGCACCAC<br>CCATGCGTACGCCAAACCGTAGAATCGACTGCCAACGCTAACGTCG<br>TCATGCACGCAG

Table S9 *List of relevant primers*

#	name	sequence	description
Primer used to produce pET-28a-MapADO with various tags			

P1	insert 1 fwd	GTTGAGGCCGTTGAGC	Amplification of DNA insert 1a and 1b
P2	insert 1 rev	CACTGGCTCCTGCGTTGCAGTAGGAGCCATGGTATATCTCCTTCTTAAAGTTAAACAAAATTATTTC	Amplification of DNA insert 1a
P3	insert 1 (with N-term StrepII) rev	AGCTTTTTCGAACTGCGGGTGGCTCCACATGGTATATCTCCTTCTTAAAGTTAAACAAAATTATTTC	Amplification of DNA insert 1b
P4	insert 2 fwd	GAGATATACCATGGCTCCTACTGCAACGC	Amplification of DNA inserts 2a and 2c
P5	insert 2 (with N-term StrepII) fwd	ATGTGGAGCCACCCGCAGTTCGAAAAAGCTCCTACTGCAACGC	Amplification of DNA insert 2b
P6	insert 2 rev	CAACTCAGCTTCCTTTCGG	Amplification of DNA inserts 2a and 2b
P7	insert 2 (with C-term StrepII) rev	CAACTCAGCTTCCTTTCGGGCTTTGTTAGCAGCCGGAAGCTTCCTATTATTTTTCGAACTGCGGGTGGCTCCAAGCGTCGGGATCGTATG	Amplification of DNA insert 2c
Primer used to produce rational-designed mutants			
P8	insert F5/6/7/9 fwd	GACTGATATTCTGCCCTCGGGATGGCCCACCGCTACTGAC	Amplification of DNA insert F5 or F6 or F7 or F9 for mutants MUT 5, 6, 7 and 9
P9	insert F5/6 rev	CTACGGTTTGGCGTACGCATGGGTGGTGCGTGTACGGGTT	Amplification of DNA insert F5 or F6 for mutants MUT 5 and 6
P10	insert F7/9rev	TTTCCTTCATGGCAAGAAAACGGCCTGCGTGCATGACGAC	Amplification of DNA insert F7 or F9 for mutants MUT 7 and 9
P11	F8_fwd	GTCGTCATGCACGCAGGCCGTTTTC	Amplification of DNA insert F8 for MUT 8
P12	F8_rev	GTTTGGCGTAAGAGCGCAATCGTGAATGAAC	Amplification of DNA insert F8 for MUT 8
P13	backbone-F5/6 fwd	CAACCCGTACACGCAC	Amplification of backbone to be combined with F5/6
P14	backbone -F5/6/7/9 rev	GTCAGTAGCGGTGGG	Amplification of backbone to be combined with F5 or F6 or F7 or F9
P15	backbone -F7/9 fwd	GTCGTCATGCACGCAG	Amplification of backbone to be combined with F7 or F9 for MUT 7 and 9
P16	BB-F8_fwd	GTTCATTCACGATTGCGCTCTTACGCCAAAC	Amplification of backbone to be combined with F8 for MUT 8

P17	BB-F8_rev	GAAAACGGCCTGCGTGCATGACGAC	Amplification of backbone to be combined with F8 for MUT 8

Preparation of *Map*ADO Variants

The genes were constructed by site-directed mutagenesis with specific primers for 5 variants (K202N, T317G, A318T, G394N, N397F), as shown in Table S10. The PCR reaction of 25 µL contained 5 ng of DNA template, 0.48 µM primer pair, 200 µM dNTPs, and 0.02 U/µL Phusion High-Fidelity DNA polymerase in 1X Phusion HF Buffer or CG buffer containing 5 % DMSO. The PCR reactions were started with an initial denaturation at 98°C for 60 seconds. Subsequently, 30 cycles of denaturation at 98°C for 10 seconds, annealing at 60-70°C for 30 seconds, and extension at 72°C for 3 minutes were carried out, followed by a final extension at 72°C for 10 minutes. The PCR products were treated with *Dpn*I at 37°C for 14 h to digest the methylated template DNA. The PCR products were then transformed into *E. coli* TOP 10 F' competent cells by electroporation. The transformed cells were spread on an LB agar plate containing 100 µg/mL Ampicillin and incubated at 37°C 16 h. A single colony from each plate was grown and the DNA plasmids were extracted and verified by DNA sequencing. *E. coli* BL21(DE3) was transformed with the verified plasmid of each ADO variant to express protein according to the protocol described above.

***Table S10**: List of primers used for site-directed mutagenesis. Mutated amino acids are displayed blot.*

**Mutation**	**Sequence (5' → 3')**
K202N	Forward: ACACCCT**AAC**CAGTGCTCGGTTACCGGCAATTTGGTGGG<br>Reverse: ACCGAGCACTG**GTT**AGGGTGTGCCGTCATAGTCTTAGATGGC
T317G	Forward: TGCCCATTCAC**GGC**GCTAGCGGTTTCGAAGACGAGCAAGGGCGCATTATC<br>Reverse: AACCGCTAGC**GCC**GTGAATGGGCATCCCATTCTTCCAATGCCAGTATTTTACTTC
A318T	Forward: ATTCACACC**ACC**AGCGGTTTCGAAGACGAGCAAGGGCGCATTATCATTG<br>Reverse: TCGAAACCGCT**GGT**GGTGTGAATGGGCATCCCATTCTTCC
G394N	Forward: AGAATTCCCG**AAC**ATTGATAATCGTTTTATGGGAGTCGAGTATTCATC<br>Reverse: ACGATTATCAAT**GTT**CGGGAATTCTGAAGGCGTATCTAAGATCAC
N397F	Forward: AAATTGAT**TTT**CGTTTTATGGGAGTCGAGTATTCATCGGC<br>Reverse: TCCCATAAAACG**AAA**ATCAATTTGCGGGAATTCTGAAG

General flask expression protocol

10 mL LB media with antibiotics (final concentration 100 µg/mL Ampicillin or 50 µg/mL Kanamycin) was inoculated with a single colony or from glycerol stock and grown as the overnight culture (ONC). ONCs were incubated at 37°C/120 rpm for 16 h.

Main cultures consisting of LB media with 100 µg/mL Ampicillin were inoculated with 2 % ONC. Main cultures were incubated at 37°C/120 rpm. At an OD_{600} of 0.6 to 0.8 protein expression was induced by addition of 1 mM

IPTG and 1 mM $FeCl_2$ supplementation. For expression, the cultures were incubated at 20°C/100 rpm for 16 to 20 h.

Cells were harvested by centrifugation of the culture at 4°C/4000 rpm for 30 min in an Eppendorf 5810 R centrifuge (≤ 50 mL volumes per vessel) or at 4°C/5000 rpm for 30 min in an Avanti JXN-26 (rotor: JA-10 Fixed-Angle Rotor- 6 x 500 mL) (≤ 500 mL volumes per vessel). The pellets were washed twice with storage buffer (10 mM phosphate buffer pH 7.4) or the desired reaction buffer e.g., 100 mM TrisHCl (pH 9.5) or 20 mM HEPES (pH 8.0).

Biotransformation reactions with *Map*ADO

Biotransformations were conducted in potassium phosphate buffer (10 mM, pH 7.4) with resting cells and varying optical densities (OD_{600}). The typical OD_{600} for experiments using wild-type *Map*ADO was 10. Substrates were added as stock solutions (c_{final} x 50) dissolved in EtOH (final amount: 2 vol. %), with a final concentration up to 40 mM. Reactions were typically incubated at 30°C or 40°C on a tissue cultivation rotor or a thermoshaker. Samples were taken at various time points (5 min to 16 h), or the entire reaction was immediately extracted twice with an equal volume of EtOAc containing acetophenone (10 mM) as an internal standard. Alternatively, the reaction mixture was diluted with an equal volume of MeOH, followed by centrifugation for 20 min at 4,000 rpm in an Eppendorf 5810 R centrifuge to remove pelleted cells and other solid components. Analysis was carried out as described in HPLC-UV analysis.

Enzyme purification

Cells were harvested by centrifugation for 30 min at 5000 rpm in a precooled Avanti JXN-26 (4°C) (rotor: JA-10 Fixed-Angle Rotor- 6 x 500 mL) and washed twice with respective binding buffer or similar.

For His-tag purification was the cell pellet resuspended in binding buffer (25 mM phosphate buffer, 500 mM NaCl, 20 mM imidazole pH 7.4) and sonicated 3 times for 120 s at 70 % amplitude on ice. Cell fragments were removed by centrifugation for 30 min at 20000 rpm in a precooled Avanti JXN-26 (4°C) (rotor: JA-25.50 Fixed-Angle Rotor- 8 x 50 mL). The supernatant was filtered through a syringe filter (0.45 µm) to remove residual particles before samples were loaded on a HisTrap FF 5 mL cartridge via sample pump (flow:5 mL/min). The column was washed with 10 CV binding buffer until no absorption was detected in the flowthrough, then the enzyme was eluted with 20 CV elution buffer (25 mM phosphate buffer, 500 mM NaCl, 500 mM imidazole (pH 7.4)) with a flow of 1 mL/min and a gradient to 100 % elution buffer over 100 min. Flow-through was collected in 1 mL fractions. Fractions containing the purified enzyme were pooled, the buffer was exchanged for storage buffer (10 mM phosphate buffer pH 7.4) and samples were concentrated using Vivaspin® Centrifugal Concentrators and stored at -20°C.

For Strep-tag II purification was the cell pellet resuspended in binding buffer (100 mM TrisHCl, 150 mM NaCl, 1 mM EDTA pH 8.0) and sonicated 3 times for 120 s at 70 % amplitude on ice. Cell fragments were removed

by centrifugation for 30 min at 20000 rpm in a precooled Avanti JXN-26 (4°C) (rotor: JA-25.50 Fixed-Angle Rotor- 8 x 50 mL). The supernatant was filtered through a syringe filter (0.45µm) to remove residual particles before samples were loaded on the StrepTrap XT 5 mL cartridge via sample pump (flow:5 mL/min).
Column was washed with 10 CV binding buffer until no absorption was detected in the flowthrough, then the enzyme was eluted with 6 CV elution buffer (100 mM TrisHCl, 150 mM NaCl, 1 mM EDTA, 5 mM biotin pH 8.0) with a flow of 1 mL/min. Flow-through was collected in 1 mL fractions. Fractions containing the purified enzyme were pooled, the buffer was exchanged for storage buffer (10 mM phosphate buffer pH 7.4) and samples were concentrated using Vivaspin® Centrifugal Concentrators and stored at -20°C.

Biophysical Characterization

The hydrodynamic radius of *Map*ADO was assessed using Dynamic Light Scattering (DLS) with a Prometeus Panta instrument (Nanotemper). *Map*ADO was diluted to 0.5 mg/mL in 20 mM Tris buffer at pH 8.0, centrifuged, and loaded into dedicated capillaries (Nanotemper) in triplicate. Data were averaged and presented with standard error.

Determination of Crystal Structures

Immobilized metal affinity chromatography (IMAC) purified *Map*ADO (8 mg/mL) was thawed, centrifuged, and checked for homogeneity using DLS and size exclusion chromatography (SEC). The protein solution was spiked with the ligands 4-hydroxybenzoic acid (HBA), ortho-vanillin, or vanillin (from Merck) at a 40-fold molecular excess. The mixture was incubated for 15 minutes at room temperature (RT) and clarified by centrifugation at 15,000 × g for 10 minutes. The supernatant was used for crystallization screening via the sitting-drop vapor diffusion method, using commercially available buffer sets. Diffraction-quality crystals were obtained at room temperature under the following conditions: Co-crystal with acetate: 20% w/v polyethylene glycol (PEG) 3,350, 200 mM lithium acetate, 30% glycerol; Co-crystal with vanillin: 0.1M PCTP (PACT premier™ crystallization buffer), pH 7.0, 25% w/v PEG 1,500, 30% glycerol; Co-crystal with HBA: 20% w/v PEG 3,350, 200 mM ammonium acetate, 30% GOL; Co-crystal with ortho-vanillin: 0.2 M sodium acetate, 0.1 M sodium HEPES, pH 7.5, 25% w/v PEG 3,350.
X-ray diffraction data were collected at the P11 beamline at PETRA III (DESY, Hamburg, Germany) and the European Synchrotron Radiation Facility (ESRF, Grenoble, France). The data were indexed, integrated, and scaled using XDS and Aimless[3]. Initial phases were obtained by molecular replacement using Phaser[4], with manual model building in Coot[4]. Refinement was carried out using the CCP4 cloud and the PDB-REDO server[5,6]. The coordinates and structure factors have been deposited in the Protein Data Bank under the accession numbers PDB ID: 9G8F, 9G8A, 9G89, and 9G88 for co-structures with ortho-vanillin, HBA, vanillin, and acetate, respectively.

Homology modelling of the oxygen containing active site of *Map*ADO

The crystal structure of chain B of *Map*ADO (PDB: 9G88) was cleared from chain A, water and ligands. NOV1 (PDB: 5J54) was cleared from water and other ligand molecules. *Map*ADO and NOV1 were aligned to the metal-binding histidine residues, iron atoms and the oxygen ligand of the active sites and extracted using the PyMOL program suite (Fig S10)[7]. The oxygen molecule of NOV1 was manually copied to the iron atom of the *Map*ADO and atomic charges of the complex were calculated by single point energy calculations using the M06L[8] functional implemented in the Gaussian 16 program suite and the def2-TZVP basis set[9] for iron (high spin), the 6-311G(d,p) basis set[10] for carbon and hydrogen and the 6-311+G(d,p) basis set[11] for nitrogen and oxygen.

Mutation and docking studies

The active site (Y136A, T156A and K169A) of *Map*ADO was mutated and side chains were relaxed using the mutate_model script of the Modeller software suite[12]. Implementation of polar hydrogens, adjustment of the protonation states of the iron binding histidines and calculation of Kolman and Gasteiger charges of the receptors was performed using the AutoDockTools 1.5.7 software suite[13]. Coordinates and charges of the dioxygen atoms and the iron center was manually added using Notepad++. Size and morphology of the binding pockets was evaluated using the AutoLigand program implemented into the AutoDockTools software suite (150 points, size x,y,z = 30)[14]. Ligand structures were converted from SMILES codes into energy-minimized 3D structures using OpenBabel 3.1.1[15]. The optimized structures were prepared for docking using the prepare_ligand4 program of the AutoDockTools suite. Docking was performed using Autodock Vina (size x,y,z = 20, center x = -5.469, center y = -11.992, center z = -81.551, exhaustiveness = 50). Visualization and analysis of the results were performed using PyMol and LigPlus[16].

Removal of Fe^{2+} ion from purified *Map*ADO

Purified *Map*ADO (60 μM) was treated with metal chelating agents, EDTA or EGTA, at a fivefold molar excess (300 μM), or with 30 mg of Chelex® 100 resin. For EDTA and EGTA treatments, the enzyme solution was gently mixed at 4 °C for 20 h. Excess chelating agents and chelation complexes were removed using a PD-10 desalting column (Sephadex G-25 resin) to obtain treated *Map*ADO. For the treatment with Chelex®100 resin, the enzyme mixture was incubated at 4 °C for 20h, and subsequently centrifuged to remove the resin. Enzyme activity was assessed by incubating 0.6 mg/mL of treated or untreated *Map*ADO with 20 mM isoeugenol in metal-free buffer (10 mM potassium phosphate, pH 7.4) at 40 °C and 400 rpm for 3 h.

Inhibition experiments

Reactions contained potassium phosphate buffer (10 mM, pH 7.4), IMAC purified *Map*ADO, EtOH (2% v/v), and 10 mM of isoeugenol as the substrate, with potential inhibitors (*ortho*-isoeugenol, *ortho*-vanillin, or vanillin) in varying concentrations (10 mM, 6 mM, 3 mM). Reactions were incubated at 30°C on a tissue cultivation rotor or thermoshaker, subsequently terminated and extracted twice with an equal volume of EtOAc containing acetophenone (10 mM) as an internal standard. Analysis was carried out as described in HPLC-UV analysis.

Spectrophotometric assay for determination of kinetic parameters

Kinetic curves with isoeugenol were determined in reactions containing potassium phosphate buffer (10 mM, pH 7.4), His-tag purified enzyme (0.4 mg/mL), isoeugenol (0.05 mM to 2 mM), and EtOH (2% v/v) in a total volume of 200 µL. Reactions were carried out in triplicates of biological triplicates. The increase in absorbance, corresponding to vanillin formation, was monitored at 350 nm for 1 min at 40°C using a Synergy Mx Platereader (Biotek).

Determination of kinetic parameters by single endpoint UPLC measurements

Kinetic curves with isoeugenol were determined as endpoint measurements in reactions containing potassium phosphate buffer (10 mM, pH 7.4), IMAC purified enzyme (0.2 to 1.0 mg/mL), isoeugenol (0.02 mM to 6 mM), and EtOH (2% v/v) in a total volume of 200 µL. Reactions were carried out in technical triplicates. Reactions were stopped and extracted twice with an equal volume of EtOAc containing acetophenone (10 mM) as an internal standard after 20 min. Analysis was carried out as described in HPLC-UV analysis.

HPLC-UV analysis

Samples were analyzed using HPLC-UV on an LC-MS 2020 system equipped with a UV detector (Shimadzu). The column used was an EC 150/3 Nucleodur C18 Gravity (3 µm, Macherey-Nagel, REF 760083.30). The mobile phases were solvent A (0.1% formic acid in double-distilled water) and solvent B (acetonitrile). Detection was performed at 280 nm with the column maintained at 40°C and a flow rate of 1 mL/min. The solvent gradient was: 0-0.7 min, 10 % B; 0.7 min – 3.1 min, 10 % to 90 % B; 3.1 – 3.8 min, 90% B, 3.8 – 5.0 min, 10% B. Data were evaluated using LabSolution software (Shimadzu), and compound quantification was based on calibration curves with authentic standards.

References

(1) Yamada, M.; Okada, Y.; Yoshida, T.; Nagasawa, T. Purification, Characterization and Gene Cloning of Isoeugenol-Degrading Enzyme from Pseudomonas Putida IE27. *Arch Microbiol* **2007**, *187* (6), 511–517. https://doi.org/10.1007/s00203-007-0218-9.

(2) Ni, J.; Wu, Y. T.; Tao, F.; Peng, Y.; Xu, P. A Coenzyme-Free Biocatalyst for the Value-Added Utilization of Lignin-Derived Aromatics. *J Am Chem Soc* **2018**, *140* (47), 16001–16005. https://doi.org/10.1021/jacs.8b08177.

(3) Evans, P. R.; Murshudov, G. N. How Good Are My Data and What Is the Resolution? *Acta Crystallogr D Biol Crystallogr* **2013**, *69* (7), 1204–1214. https://doi.org/10.1107/S0907444913000061.

(4) Emsley, P.; Lohkamp, B.; Scott, W. G.; Cowtan, K. Features and Development of Coot. *Acta Crystallogr D Biol Crystallogr* **2010**, *66* (4), 486–501. https://doi.org/10.1107/S0907444910007493.

(5) Joosten, R. P.; Long, F.; Murshudov, G. N.; Perrakis, A. The PDB-REDO Server for Macromolecular Structure Model Optimization. *IUCrJ* **2014**, *1*, 213–220. https://doi.org/10.1107/S2052252514009324.

(6) Krissinel, E.; Lebedev, A. A.; Uski, V.; Ballard, C. B.; Keegan, R. M.; Kovalevskiy, O.; Nicholls, R. A.; Pannu, N. S.; Skubák, P.; Berrisford, J.; Fando, M.; Lohkamp, B.; Wojdyr, M.; Simpkin, A. J.; Thomas, J. M. H.; Oliver, C.; Vonrhein, C.; Chojnowski, G.; Basle, A.; Purkiss, A.; Isupov, M. N.; Mcnicholas, S.; Lowe, E.; Trivinõ, J.; Cowtan, K.; Agirre, J.; Rigden, D. J.; Uson, I.; Lamzin, V.; Tews, I.; Bricogne, G.; Leslie, A. G. W.; Brown, D. G.; Antonyuk, S. CCP4 Cloud for Structure Determination and Project Management in Macromolecular Crystallography. *Acta Crystallogr D Struct Biol* **2022**, *78* (Pt 9), 1079–1089. https://doi.org/10.1107/S2059798322007987.

(7) Schrödinger L.; DeLano, W. PyMOL. **2020**. https://doi.org/http://www.pymol.org/pymol.

(8) Zhao, Y.; Truhlar, D. G. A New Local Density Functional for Main-Group Thermochemistry, Transition Metal Bonding, Thermochemical Kinetics, and Noncovalent Interactions. *J Chem Phys* **2006**, *125* (19), 194101. https://doi.org/10.1063/1.2370993.

(9) Weigend, F.; Ahlrichs, R. Balanced Basis Sets of Split Valence, Triple Zeta Valence and Quadruple Zeta Valence Quality for H to Rn: Design and Assessment of Accuracy. *Physical Chemistry Chemical Physics* **2005**, *7* (18), 3297–3305. https://doi.org/10.1039/b508541a.

(10) Krishnan, R.; Binkley, J. S.; Seeger, R.; Pople, J. A. Self-consistent Molecular Orbital Methods. XX. A Basis Set for Correlated Wave Functions. *J Chem Phys* **1980**, *72* (1), 650–654. https://doi.org/10.1063/1.438955.

(11) Foreman, J. B.; Head-Gordon, M.; Pople, J. A.; Frisch, M. J. *Toward a Systematic Molecular Orbital Theory for Excited States*; Pergamon Press, 1973; Vol. 58. https://doi.org/doi:10.1021/j100180a030.

(12) Sali, A.; Blundell, T. L. Comparative Protein Modelling by Satisfaction of Spatial Restraints. *J Mol Biol* **1993**, No. 234, 779–815. https://doi.org/10.1006/jmbi.1993.1626.

(13) Morris, G. M.; Ruth, H.; Lindstrom, W.; Sanner, M. F.; Belew, R. K.; Goodsell, D. S.; Olson, A. J. Software News and Updates AutoDock4 and AutoDockTools4: Automated Docking with Selective Receptor Flexibility. *J Comput Chem* **2009**, *30* (16), 2785–2791. https://doi.org/10.1002/jcc.21256.

(14) Harris, R.; Olson, A. J.; Goodsell, D. S. Automated Prediction of Ligand-Binding Sites in Proteins. *Proteins: Structure, Function and Genetics* **2008**, *70* (4), 1506–1517. https://doi.org/10.1002/prot.21645.

(15) O'Boyle, N. M.; Banck, M.; James, C. A.; Morley, C.; Vandermeersch, T.; Hutchison, G. R. Open Babel: An Open Chemical Toolbox. *J Cheminform* **2011**, *3* (10). https://doi.org/10.1186/1758-2946-3-33.

(16) Laskowski, R. A.; Swindells, M. B. LigPlot+: Multiple Ligand-Protein Interaction Diagrams for Drug Discovery. *J Chem Inf Model* **2011**, *51* (10), 2778–2786. https://doi.org/10.1021/ci200227u.
